# A Computational Model of Bacterial Population Dynamics in Gastrointestinal *Yersinia enterocolitica* Infections in Mice

**DOI:** 10.3390/biology11020297

**Published:** 2022-02-12

**Authors:** Janina K. Geißert, Erwin Bohn, Reihaneh Mostolizadeh, Andreas Dräger, Ingo B. Autenrieth, Sina Beier, Oliver Deusch, Alina Renz, Martin Eichner, Monika S. Schütz

**Affiliations:** 1 Institute for Medical Microbiology and Hygiene, University Hospital Tübingen, Elfriede-Aulhorn-Str. 6, 72076 Tübingen, Germany; janina.geissert@med.uni-tuebingen.de (J.K.G.); erwin.bohn@med.uni-tuebingen.de (E.B.); laed@med.uni-heidelberg.de (I.B.A.); 2 Computational Systems Biology of Infections and Antimicrobial-Resistant Pathogens, Institute for Bioinformatics and Medical Informatics (IBMI), University of Tübingen, Sand 14, 72076 Tübingen, Germany; alina.renz@uni-tuebingen.de; 3 Department of Computer Science, University of Tübingen, Sand 14, 72076 Tübingen, Germany; sb2534@cam.ac.uk; 4 German Center for Infection Research (DZIF), Partner Site Tübingen, 72076 Tübingen, Germany; 5 Cluster of Excellence ‘Controlling Microbes to Fight Infections’, University of Tübingen, 72076 Tübingen, Germany; 6 CEMET GmbH, Eisenbahnstr. 63, 72072 Tübingen, Germany; oliver.deusch@googlemail.com; 7 Epimos GmbH, Frühlingstraße 2, 97653 Bischofsheim in der Rhön, Germany; martin.eichner@uni-tuebingen.de; 8 Institute for Clinical Epidemiology and Applied Biometry, University of Tübingen, Silcherstraße 5, 72076 Tübingen, Germany

**Keywords:** infection, systems biology, computational modeling, population dynamics, gastrointestinal infection, ordinary differential equations, parameter estimation, *Yersinia enterocolitica*

## Abstract

**Simple Summary:**

Computational modeling of bacterial infection is an attractive way to simulate infection scenarios. In the long-term, such models could be used to identify factors that make individuals more susceptible to infection, or how interference with bacterial growth influences the course of bacterial infection. This study used different mouse infection models (immunocompetent, lacking a microbiota, and immunodeficient models) to develop a basic mathematical model of a *Yersinia enterocolitica* gastrointestinal infection. We showed that our model can reflect our findings derived from mouse infections, and we demonstrated how crucial the exact knowledge about parameters influencing the population dynamics is. Still, we think that computational models will be of great value in the future; however, to foster the development of more complex models, we propose the broad implementation of the interdisciplinary training of mathematicians and biologists.

**Abstract:**

The complex interplay of a pathogen with its virulence and fitness factors, the host’s immune response, and the endogenous microbiome determine the course and outcome of gastrointestinal infection. The expansion of a pathogen within the gastrointestinal tract implies an increased risk of developing severe systemic infections, especially in dysbiotic or immunocompromised individuals. We developed a mechanistic computational model that calculates and simulates such scenarios, based on an ordinary differential equation system, to explain the bacterial population dynamics during gastrointestinal infection. For implementing the model and estimating its parameters, oral mouse infection experiments with the enteropathogen, *Yersinia enterocolitica* (Ye), were carried out. Our model accounts for specific pathogen characteristics and is intended to reflect scenarios where colonization resistance, mediated by the endogenous microbiome, is lacking, or where the immune response is partially impaired. Fitting our data from experimental mouse infections, we can justify our model setup and deduce cues for further model improvement. The model is freely available, in SBML format, from the BioModels Database under the accession number MODEL2002070001.

## 1. Introduction

The gastrointestinal microbiome provides resistance to pathogen colonization and infection by contributing to the development of the host immune system [1,2] and conferring colonization resistance (CR) [3], as well as the direct competition of a pathogen with members or compounds produced by the microbiota [4]. Consequently, the disruption of the endogenous microbiome results in an increased susceptibility to infection [5,6,7] and can be induced by several means, including the host’s inflammatory response to infection or antibiosis [8,9]. Together with the physical barriers provided by the epithelial surface lining and the adherent mucus, several effectors of the intestinal immune system prevent the entry and systemic spread of pathogenic bacteria present in the gut lumen, while allowing for the existence of a complex microbiome. Several measures of the immune system contribute to this involved process; amongst them are the production of secretory IgA, the release of antimicrobial peptides (AMP), the expression of pro-inflammatory cytokines, and the recruitment of, e.g., neutrophils [10,11].

Gastrointestinal infection is a frequent disease that causes significant morbidity and a high economic burden [12,13]. Being self-resolving in most cases, the symptomatic treatment (e.g., rehydration) is sufficient for otherwise healthy individuals. In contrast, gastrointestinal tract (GIT) infection can cause high morbidity, and even fatal diseases, in healthcare settings and specific populations, such as newborns, the elderly, and immunocompromised individuals. According to the OECD Health Report 2016–2017, approximately 9% of healthcare-associated infections were related to the GIT (OECD/European Union Paris/European Union, (2018)). Therefore, understanding the underlying mechanisms and identifying the crucial factors for a mild or severe course of infection is highly desirable.

The Gram-negative, facultative anaerobe pathogen, *Yersinia enterocolitica* (Ye), has been used previously to study pathogen interactions in the GIT within the host to identify virulence factors that are crucial for the successful colonization of Ye and to find out how individual virulence factors interact with the host [14,15,16,17,18,19,20,21,22,23]. Within the small intestine (SI), Ye can adhere to, and invade, the intestinal epithelial lining, mainly via the adhesins, *Yersinia* adhesin A (YadA) [24] and Invasin [25,26,27,28]. Upon attachment, Ye can engage its type-three secretion system (T3SS). The T3SS facilitates the injection of effector proteins (*Yersinia* outer proteins, Yops), which contribute to the immune evasion and the establishment of infection [28,29]. Ye can invade the Peyer’s patches (PP) to form abscesses and disseminate into peripheral lymphatic tissues [30,31]. YadA is the most crucial individual virulence factor of Ye. It was shown that a YadA-deficient strain was impaired in the colonization and systemic spread in mouse infection [19]. The first line of host defense against invading Ye is a massive infiltration of phagocytic cells. However, Ye can counteract phagocytosis via its T3SS [28,29]. This Ye virulence trait seems crucial to evading phagocytic killing in vivo [28]. Together, both the T3SS and YadA, presumably, contribute to the efficient colonization of the intestinal tract, while Ye, at the same time, induces an inflammatory response that might account for a reduction in the density and complexity of the commensal microbiome [8,9].

In recent years, several models were developed to mirror bacterial gastrointestinal infections [32,33,34,35,36,37], viral infections at epithelial sites [38], and inflammatory disorders, such as IBD [39,40]. This study aims to utilize quantitative data to understand the mechanisms driving such gastrointestinal diseases. To this end, we derived a mechanistic model of gastrointestinal infection based on ordinary differential equations (ODEs) as a method to identify parameters that are decisive for the infection course. In contrast to previous models, our model allows us to simultaneously modulate the virulence and growth rate of the causative pathogen, as well as modulating of underlying host conditions, such as the immune competence and the presence of a microbiome. Therefore, it may contribute to extending our knowledge about their role in the course of infection. We used experimental data from a basic mouse model of infection setting to parametrize our model, experimentally determine several accessible parameter values, and justify our model design by fitting experimental data with distinct changed parameters. In sum, we want our computational model to provide hints about which parameters are decisive for the infection course, as well as serving as a hypothesis generator of how Ye–host–microbiota interactions take place in vivo, and, therefore, use this as a starting point for the development of more elaborate models of gastrointestinal infection. 

## 2. Materials and Methods

### 2.1. Bacterial Strains and Growth Conditions

The Ye wt and mutant strains used in this study are listed in Supplementary Table S2. All strains were cultured overnight at 27 °C in a Luria Bertani broth (LB). All selective antibiotics, such as nalidixic acid (10 µg/mL), kanamycin (50 µg/mL), spectinomycin (100 µg/mL), and chloramphenicol (25 µg/mL) (all Sigma-Aldrich) were supplemented in combinations according to the indicated resistances (Table S2). For the preparation of bacterial suspensions for oral infection, overnight cultures were diluted and subcultured for 3 h at 27 °C. Bacteria were then washed once with Dulbecco’s phosphate-buffered saline (DPBS, Gibco, Thermo Fisher Scientific, Waltham, MA, USA), and the OD_600_ was determined to prepare the desired inoculum.

### 2.2. Generation of Ye Strains Containing Different Antibiotic Selection Markers

A chloramphenicol resistance cassette, derived from pASK IBA4C (IBA Lifesciences, Göttingen, Germany), was chromosomally introduced into the YenI locus of the Ye WAC strain to discriminate between the Ye wt and the Ye YadA0 or the T3SS-deficient strain (T3S0). The YenI gene encodes for a Ye-specific restriction-modification system, the interruption of which allows for a higher efficiency of genetic manipulations [41,42]. The resistance cassette was inserted by homologous recombination using the suicide plasmid pSB890Y, as described previously [23], and the insertion was verified by PCR, antibiotic resistance testing, and sequencing. Finally, the respective virulence plasmids were re-transformed into Ye WAC Cm^R^. All plasmids and primers used for the insertion of selection markers are listed in the Supplementary Tables S2 and S3.

### 2.3. Animal Handling

Ethics statement: All animal infection experiments were approved by the regional authority of the state of Baden-Württemberg in Tübingen (permission number H2/15). Female C57BL/6J OlaHsd mice were purchased from Envigo (Horst, Netherlands). MyD88-deficient mice (*MyD88*^−/−^) with a C57BL/6J genetic background were obtained from a local breeding colony (breeding pairs were purchased from Jackson Laboratories, Bar Harbor, ME, USA). Animals were housed in the animal facility of the University Hospital Tübingen under specific pathogen-free (SPF) conditions. Germ-free (GF) animals were bred in the germ-free core facility of the University Hospital Tübingen or were provided by the Institute for Laboratory Animal Science (Hannover Medical School, Hannover, Germany). All animals were housed in individually ventilated cages in groups of five animals and were supplied with autoclaved food and drinking water ad libitum. Infection experiments were performed with female mice at 6–10 weeks of age.

### 2.4. Oral Mouse Infection

Prior to the intragastric administration of bacteria, mice were deprived of food and water for 3–4 h. For oral coinfection experiments, animals were infected with a 1:1 mixture of 2.5 × 10^8^ CFU of Ye wt and Ye YadA0, or Ye T3S0, respectively. Upon the oral coinfection, SPF wild-type and GF mice were sacrificed at time points indicated within the figures describing the results of individual experiments. The *MyD88*^−/−^ mice were infected for two days only because of the expected rapid systemic spread in these immunocompromised animals. Oral infections for subsequent RNA analyses from small intestinal mucosal scrapings were performed for two days.

### 2.5. Determination of Bacterial Load from Feces

Fresh fecal pellets were collected after the manual stimulation of individual mice, were weighed using a high-precision laboratory scale, and were resuspended in 500 µL of sterile DPBS. Pellets were homogenized, serially diluted with DPBS, plated on selective agar plates, and incubated at 27 °C for 48 h. Afterward, colonies were counted, and the CFUs per gram of feces were calculated.

### 2.6. Calculation of Competitive Indices in Mixed Infections

Competitive indices (CI) from fecal and tissue samples were calculated as the CFU output of the Ye mutant/Ye wild-type strains, divided by the input (initial oral inoculum) of these strains (CFU Ye mutant strain input/CFU Ye wild-type strain input). The output was determined in the individual experiments, as described above. The initial oral inocula (=the input) were verified by serial dilution and their subsequent plating on LB with appropriate antibiotics. A CI, with a logarithmic value of zero, indicates the identical fitness of the wild-type and the mutant strains, while a negative CI indicates that the mutant strain is impaired in colonization [43].

### 2.7. 16S rRNA Sequencing from SI Luminal Samples

For the analysis of the microbiota composition within the SI of mice, and to assess changes in microbiota composition upon infection with Ye, mice were initially co-housed for ten days. After the oral infection with Ye, as described before, or after the gavage of the same volume of DPBS, mice were sacrificed at the indicated points in time. The entire GIT was dissected, and the SI was removed. The intestinal contents were isolated by gently squeezing them into tubes using sterile forceps. After that, the samples were immediately snap-frozen and stored at −80 °C until DNA isolation. DNA was extracted, as described in the International Human Microbiome Project Standard (IHMS) Protocol H (http://www.human-microbiome.org/index.php?id=Sop&num=007, accessed date: 8 February 2022) [44]. The library preparation and 16S rRNA amplicon sequencing were performed by the CeMet Company (Tübingen) using variable regions v3-v4. Paired-end sequencing was performed on the Illumina MiSeq platform (MiSeq Reagent Kit v3) with 600 cycles. Raw read quality control was done using the FastQC tool (https://www.bioinformatics.babraham.ac.uk/projects/fastqc/, accessed date: 8 February 2022 [45]). To this end, reads were merged, and quality filtering was performed using USEARCH [46]. The taxonomy data annotation of sequences was done by comparison against the National Center for Biotechnology Information (NCBI) bacterial 16S rRNA database using MALT [47]. Abundance tables at the taxonomic rank of interest were generated using MEGAN6 [48] and were further analyzed using the software R (https://www.r-project.org, accessed date: 8 February 2022 [49]). Before applying statistical analyses, all samples were normalized to 14,947 reads (the lowest number of reads across all samples) using the tool rrarefy, which is part of the VEGAN package [50]. The VEGAN package diversity function was used to calculate the Shannon diversity. An unpaired Wilcoxon sum-rank test determined the significant differences between groups. Vegsdist and prcomp (part of the VEGAN package) were used to perform a principal component analysis (PCA) on the Bray–Curtis dissimilarities. For the generation of graphical output, ggplot2 [51] was employed. The 16S rRNA sequencing data will be published at the European Nucleotide Archive database (accession number: PRJEB50711). See Supplementary Figure S1 for details.

### 2.8. Isolation of RNA from Gut Mucosal Scrapings

For the isolation of the total RNA from gut mucosal scrapings, five mice per group, harboring either an SPF microbiome or GF, and the genetic backgrounds indicated earlier, were infected with a 1:1 mixture of 2.5 × 10^8^ CFU of Ye wt, and Ye T3S0. As controls, five mice of each colonization state and genetic background were orally administered with 100 µL DPBS instead of bacterial suspensions. Two days after infection, the mice were sacrificed, and the distal 10 cm of the small intestine was dissected and shortly incubated in RNAlater (Qiagen, Hilden, Germany). Then, the tissue was flushed with ice-cold DPBS to remove the fecal content and was opened longitudinally on ice using scissors. After the removal of the residual feces by flushing again with ice cold DPBS, the mucosa was scraped off with the blunt side of a scalpel and was incubated overnight in RNA at 4 °C. The RNA was then removed, and scrapings were homogenized in a TRI-Reagent (Zymo Research, Freiburg, Germany) by rinsing them successively through syringe needles with decreasing diameters. The remaining cell debris was removed by centrifugation, and the supernatants were finally used for the RNA purification using the DirectZol RNA Miniprep Plus Kit (Zymo Research) according to the manufacturer’s protocol. This protocol included a step for the removal of genomic DNA. The resulting RNA was quantified using a Nanodrop photometer (Thermo Fisher Scientific, Waltham, MA, USA) and the integrity was confirmed by agarose gel electrophoresis.

### 2.9. Quantification of Immune Parameters by Quantitative Real-Time-PCR (qRT-PCR)

The relative mRNA levels of target genes were determined using qRT-PCR. After an additional treatment for the removal of genomic DNA included in the QuantiTect reverse transcription kit (Qiagen), mRNA was reverse transcribed according to the manufacturer’s protocol using 1 µg of RNA as the input for a 20 µL reaction. For the subsequent qRT-PCR, the TaqMan gene expression master mix (Applied Biosystems; all assays are listed in Table S3) was used with thermal cycling conditions, according to the manufacturer’s protocol. Absolute quantifications were performed on a LightCycler 480 instrument (Roche) using the LightCycler 480 Software 1.5. The relative gene expression levels of target genes to the reference gene *beta-glucuronidase* (accession number, AI747421) [52] were determined to apply a kinetic PCR efficiency correction, according to the method of Pfaffl [53], and were normalized to the expression levels of the uninfected SPF-colonized mice.

### 2.10. Determination of the Distribution of Ye along the Mouse GIT

To determine the ratio of Ye and the cultivable commensal bacteria in the different compartments of the GIT, three mice were orally infected with 5 × 10^8^ CFU of the Ye wt strain. Seven days after infection, mice were sacrificed, and the gut was dissected. A piece of tissue, 1 cm in length, that was directly adjacent to the stomach was removed, and the residual small intestine was split into three pieces of equal length: a proximal part (SI 1), a middle part (SI 2), and a distal part (SI 3). Additionally, the cecum and the colon were dissected. The contents of the three small intestinal pieces, the cecum, and the colon were isolated by gently squeezing them into tubes using sterile forceps. For each compartment, the CFU per gram of intestinal content was determined, as described above for feces, using selective agar to determine the Ye CFUs, as well as the brain-heart infusion agar (BHI; incubated in anaerobic pots) for the determination of the approximate number of cultivable commensal bacteria.

### 2.11. Systemic Administration of Gentamicin for the Cleansing of a Potential Niche Colonized by Ye

To investigate the existence of extra-luminal Ye that drained into the lumen of the SI, we tested if the systemic administration of an antibiotic that can kill Ye, but that is not able to enter the lumen of the GIT, might reduce the Ye burden in feces. To this end, 14 mice were coinfected with Ye wt and Ye YadA0 for two days. At this point in time, we assumed the successful colonization of a niche and a high bacterial burden in the feces. Mice were then split into two groups, of which one was administered intraperitoneally with 40 mg/kg gentamicin (Ratiopharm, Ulm, Germany) in 200 µL of 0.9% sterile NaCl (Braun, Kronberg, Germany). The other group was administered sterile saline only. Ye CFUs were determined from the feces of mice before the gentamicin/saline administration (i.e., on 2 dpi) and one day after treatment (i.e., on 3 dpi), as described above. The 3 dpi mice were sacrificed, and Ye CFUs were additionally determined from Peyer’s patches.

### 2.12. Determination of GIT Passage of Time

SPF C57BL/6 wild-type or *MyD88^−/−^* mice, as well as GF wild-type mice (2 mice/group), were orally challenged with 100 µL DPBS containing 1 × 10^9^ fluorescent polystyrene beads (1 µm) (Thermo Fisher), plus 5 × 10^8^ CFU Ye wt, in order to simulate infection conditions. After the gavage, fecal pellets were collected hourly over 24 h, weighed, snap-frozen, and stored at −20 °C, until analyses. Next, samples were homogenized in 1 mL of PBS, and debris was removed by a centrifugation step of 20 min with 50× *g* [54]. To determine the number of fluorescent events per gram of feces, the resulting supernatant was spiked with a defined number of compensation beads (BD biosciences, Heidelberg, Germany) to determine the number of fluorescent beads in a defined volume by flow cytometry. The cumulated bead-hours were then calculated by multiplying the number of beads detected by the time spent in the gut until excretion. The mean residence time per bead was finally calculated by dividing the number of summarized events/g feces by the total bead-hours.

### 2.13. Determination of the Water Content of the SI Content and Fecal Pellets

Three mice each, with either SPF or GF microbiota, were used for this experiment. Two to five fecal pellets were collected before the dissection of the GI tract to determine the water content. Then mice were sacrificed, and the entire GI tract was removed. Afterward, the stomach was discarded, and the small intestine was cut into two pieces of comparable length. The cecum and colon were dissected. All the pieces and the fecal pellets were placed into individual, weighed Petri dishes. The wet weight of all samples was determined. The SI pieces, the cecum, and the colon were then cut open, and the content was scratched off and transferred into a Petri dish. The remaining emptied tissue was removed and weighed again, and the wet weight of the contents was determined. The Petri dishes were placed into an incubator without lids, and the material was dried overnight at 65 °C. Then, all samples were weighed again to determine the dry weight. Finally, the total water content was calculated by subtracting the dry weight from the wet weight.

### 2.14. Calculation of the Thickening Factor for SPF and GF Mice

Our model predicted the dynamics of the number of *Yersinia* (i.e., CFU) within the SI, whereas our experimental observations were based on colony counts derived from the plating of fecal pellets (log_10_ CFU per g of feces). To align model output to our experimental data, we determined the mean percentage of water in different sections of the gastrointestinal tracts of SPF or GF mice. We considered that the small intestinal content is massively concentrated to be excreted as a solid fecal pellet. Based on these data, we calculated a “thickening factor.” The content of the SI of SPF mice had a reasonably different percentage of water (77%) compared to that of the fecal pellets (29%; Table S1). Therefore, the model predictions were multiplied with a correction factor to relate the model output to the laboratory observations. This factor is obtained by dividing the product of 1 g of fecal pellets and its content of solid matter (100–29%) by the product of the mass of the SI content of SPF mice (which is about 2.3 g) and its content of solid matter (100–77%); i.e., the factor is (1 g × 71%)/(2.3 g × 23%) ≈ 1.3. Thus, our model output needed to be multiplied by 1.3 before it can be compared with experimentally determined CFU levels for SPF mice. GF mice differ in several aspects, compared to SPF mice. They have a massively enlarged intestine (we measured the mass of intestinal contents to be about 10 g). The average water content of the fecal pellets is 49% in these mice. Using the same calculation as above, we obtain a multiplication factor of (1 g × 51%)/(10 g × 23%) ≈ 0.2 for GF mice.

### 2.15. Alignment of Model Simulation and Lab Observation Time

We determined the passage of time of the GIT to take, on average, 4 h in SPF wild-type mice, 5.5 h in *MyD88^−/−^* mice, and 12 h in GF mice (Figure S5). Assuming a 1 h passage time in the stomach and 1 h in the colon, this leaves a sojourn time of 2 h (3.5 h in *MyD88^−/−^* mice and 10 h in GF mice) in the SI, in which Ye are assumed to multiply. Our model only describes what is happening in the SI, starting when Ye leaves the stomach. This corresponds to one hour post-infection (hpi). An additional hour is needed for the colon passage until the CFUs can be counted. Thus, the observation in the laboratory at, e.g., 24 h after oral infection must be compared with the model results after 22 h of model simulation. This time shift of 2 h is taken into consideration whenever modeling results and experimental data are compared.

### 2.16. Parameter Optimization

We derived a seven-dimensional ordinary differential-equation system, describing Ye population dynamics, with seven unknown parameter values in SPF mice and eight unknown parameters in GF and *MyD88^−/−^* mice. For the estimation of the unknown model parameters in this study, the performances of different optimization algorithms were compared systematically. We applied the steepest descent method, whose convergence depends on gradient-like Lipschitz conditions for a finite number of dimensions [55,56] or an infinite number of dimensions [57,58,59,60,61]. However, the results were not robust with this approach, and the method has a slow prediction convergence. We subsequently used the particle swarm optimization (PSO) method [62], which is particularly suited to solve problems where the optimal solution is a point in the multi-dimensional space of the parameter (real-valued optimization) [63]. This method, however, easily falls into premature solutions, leading to low accuracy.

The method that turned out to be most appropriate for the model system, and was finally applied, was the maximum log-likelihood method. This method was modified to an integrated likelihood approach to account for experimental data points which had values below the limit of detection (LOD). Experimental values below the LOD of the bacterial load per g feces, in a given volume of fecal suspension, were set to log_10_ CFU/g feces of 2.05 (corresponds to the LOD in the experimental setting in C57BL/6J wild-type SPF). These values were estimated by applying a fitness function and solving the optimization problem using the maximum log-likelihood method. The objective function was defined to minimize the Euclidean distance between the measurements and model output.

To collect repeated measurements from each mouse and to avoid using each point in time as an independent measurement, we calculated the median of repeated measurements for each point in time. To ensure that the algorithm converged to the global minimum of our objective function, we ran the algorithm 100 times with multiple starting points and selected the resulting parameter values that corresponded to the minimum value obtained for the objective function. We solved the optimization problems using multi-start local optimization. This approach has been shown to perform well in systems and computational biology [64]. We assumed the standard deviation as an estimated parameter in the maximum likelihood method. Therefore, the standard deviation was estimated with each run with other unknown model parameters. We applied this method to determine whether the estimated parameters were the best fit for our model. This maximum likelihood approach, combined with the iteration count, increased the chance of convergence [65]. Eventually, the optimization was implemented using the bound-constrained optimization package FMINSEARCHBND in MATLAB 2019 (MathWorks Inc., Natick, MA, USA).

## 3. Results

We first conducted laboratory experiments to generate experimental datasets and we summarized the current knowledge of infection scenarios with Ye in (i) immunocompetent hosts with complex microbiomes, (ii) immunocompetent hosts with no microbiome, and (iii) immunodeficient hosts with the complex microbiome. The initially generated experimental dataset described the Ye infection scenario (i). We further elaborated on the presumptions for the mathematical description and introduced the ordinary differential expressions describing the mathematical model. This dynamic population model was then validated using parameter estimation, parameter fitting, and parameter sensitivity analyses based on the generated experimental dataset. Additional experimental datasets were generated in laboratory experiments to cover the infection scenarios (ii) and (iii). These datasets were eventually used to refine the dynamic population model.

### 3.1. Generation of Experimental Datasets to Generate a Dynamic Population Model

#### 3.1.1. Ye Population Dynamics Are Investigated in the Presence of a Complex Microbiome and an Intact Host Immune Response

**Figure 1:**
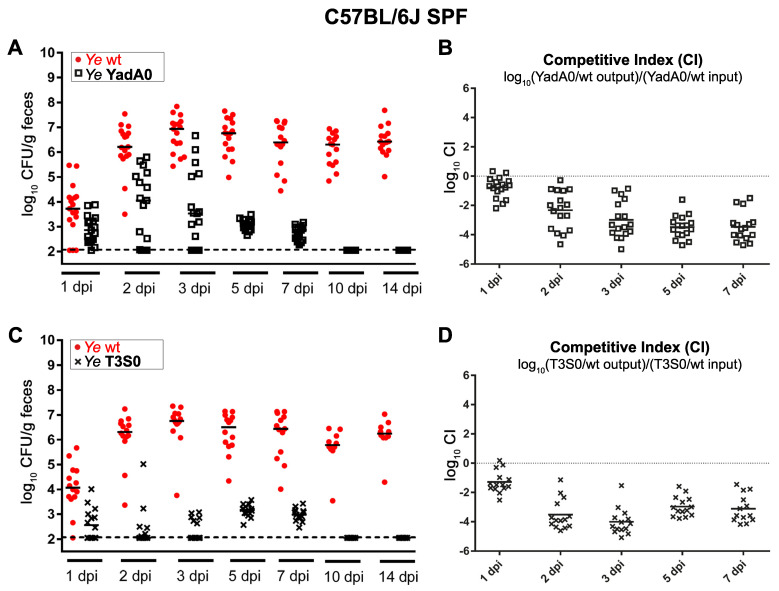
Ye population dynamics during coinfection of SPF-colonized mice. (**A**) Colony-forming units (CFU) in feces of individual animals (*n* = 14) at different points in time (days post-infection; dpi) and the median after oral 1:1 co-infection of C57BL/6J SPF mice with a Ye wild-type (wt) strain and an attenuated mutant strain lacking the *Yersinia* adhesin A (Ye YadA0). The limit of detection is indicated by a dashed line. (**B**) The competitive index (CI) of the Ye wt:Ye YadA0 coinfection was calculated as indicated. A negative CI is indicative of an attenuation of the mutant strain. (**C**) CFU in feces of individual mice after co-infection with Ye wt, and a mutant impaired in type-three secretion (Ye T3S0). (**D**) CI of the Ye at Ye wt:Ye T3S0 c-infection.

The starting point of our study was to generate an experimental dataset to develop our model of bacterial population dynamics in gastrointestinal Ye infection. We used an immunocompetent host harboring a complex microbiome. The C57BL/6J wild-type (wt) and specific pathogen-free (SPF) mice were infected with a 1:1 mixture of the Ye wt and either a Ye YadA0 mutant strain that lacked the adhesin YadA, or a mutant strain impaired in type-three secretions (T3S0). Although this is seemingly counterintuitive, we consciously decided to use this coinfection setting to create a situation where the mutant and the wild-type strains faced comparable immune responses. Single infections with Ye YadA0 or Ye T3S0 would have raised an immune response that was different compared to each other, and to that of the wild-type strain, making the reasonable comparison of infection scenarios questionable. As a high pathogen burden within the GIT increases the likeliness of systemic dissemination, the CFUs (colony-forming units) in feces may serve as an indicator to identify individuals that have an increased risk of developing a systemic infection. Thus, we determined the bacterial counts of Ye wt and the coinfected mutant strains in feces by plating them on selective media (Figure 1A,C). We found that the Ye wt strain was able to stably colonize the GIT of all animals over the entire observation period of 14 days. In contrast, the bacterial counts of both the YadA0 and the T3S0 mutant strains never reached the Ye wt level and dropped below our limit of detection at ten days post-infection (dpi). The competitive indices (Figure 1B,D) clearly show the reduced virulence of Ye YadA0 and Ye T3S0 compared to the wt.

The most striking difference between the YadA0 and T3S0 coinfection was that the bacterial counts of the Ye T3S0 strain peaked later and at considerably lower levels compared to Ye wt and compared to that of the YadA0 mutant strain.

In summary, these data indicate that the pleiotropic functions of YadA and, even more compelling, the effector functions mediated by the T3SS seem to be crucial for the effective immune evasion and colonization of the GIT in the presence of a complex microbiome and an immunocompetent host. This effect has been shown in coinfections for the first time, but has been demonstrated previously in oral single infections using the YadA deficient strain, and in coinfections with a strain lacking the single effector protein, Yop H [14,66].

#### 3.1.2. The Integration of Experimental Mouse Infection Data, Specific Parameters Determined in Wet-Lab Experiments, and Published Knowledge Are Used to Generate a Conclusive View of Ye Mouse Infection

**Figure 2:**
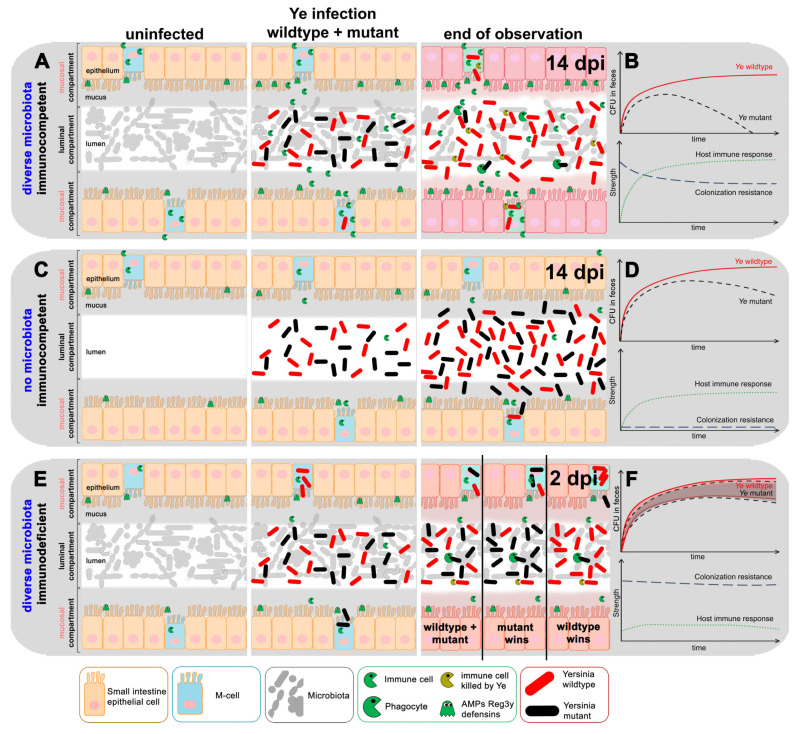
Schematic overview of the presumed infection progression after coinfection of different mouse models with Ye wt and mutant strains. (**A**) Scheme of the small intestine of SPF-colonized C57BL/6J wild-type mice during homeostasis (left), after initial disturbance (mid), and expected outcome after co-infection with a 1:1 mixture of Ye wt and an attenuated mutant strain. Initially, the gut lumen in SPF mice is densely colonized with a complex microbiota. Ye infection, associated with an infiltration of microfold cells (M-cells) mainly conducted by the wt strain, leads to an unspecific antimicrobial immune response accompanied by the release of phagocytic cells into the gut lumen and augmented expression of antimicrobial proteins (AMPs, Reg3*γ*, defensins) by epithelial cells. Both the antimicrobial response and inflammation affect at least parts of the microbiota and reduce its complexity and density. Whereas Ye wt can counteract phagocytosis by injection of effectors into immune cells, thereby killing them, the Ye mutant strain is more susceptible to phagocytosis and killing by immune cells and, thus, is finally outcompeted 14 days after infection onset. (**B**) Schematic overview of expected Ye wt and mutant CFU in feces during the infection course (upper diagram) and the presumed strength of host immune response and colonization resistance (CR; bottom diagram). (**C**) In germ-free (GF) mice that lack a microbiota that confers CR and harbor an immature immune system, Ye wt and mutant strains are both able to colonize the gut lumen and do not necessarily need to enter a site near the mucosa to colonize the gut effectively. This leads to weak antimicrobial responses that Ye can cope with, without the necessity to possess specific virulence traits (such as YadA or a functional T3SS). This results in comparable numbers of wt and mutant strains at the end of the observation period. (**D**) Presumed CFUs of Ye wt and mutant strain in feces of GF mice (upper diagram). The immune responses in GF animals are less potent as compared to C57BL/6J wild-type mice, while microbial CR is absent (bottom diagram). (**E**) In SPF-colonized *MyD88*^−/−^ mice, we assume that the strongly limited immune reaction does not significantly affect the CR that is mediated by the endogenous microbiota. This will, presumably, result in a lower overall Ye cell count in the gut compared to the SPF wild-type and GF mice. The immune deficiency entails an almost contingent infection outcome (right panel), resulting in either comparable numbers of the Ye wt and the mutant strains, or one of the strains becoming more abundant at two days after infection. Please note that the infection course in the *MyD88*^−/−^ mice can only be monitored for a shorter period due to adherence to animal welfare regulations. (**F**) The presumed coincidental CFU development in feces is illustrated by overlapping, shaded areas (upper diagram). Limited immune responses reduce CR to a low level (bottom diagram); dpi = days post-infection.

We summarized our current conception of the gastrointestinal Ye infection of a healthy, immunocompetent host, colonized with a complex microbiome by a tangible visualization (Figure 2A). The figure includes the presumed development of CFU in feces and the strength of CR and the immune response of the host over time (Figure 2B). We also tried to devise scenarios for a host lacking a microbiome (Figure 2C,D) and an immunocompromised host (Figure 2E,F). We depicted all entities that should be considered in our model, and the events taking place during homeostasis (before infection), upon the initiation of coinfection, and at a later time point after coinfection. During homeostasis in SPF wild-type animals (Figure 2A, left side “Homeostasis”), basal levels of AMPs, produced by specialized epithelial cells that are present in high concentrations in the dense mucus layer, restrict the access of microbiota components to the epithelium, thereby contributing to the maintenance of the steady-state [67,68,69]. Upon coinfection with the Ye wt and a mutant strain (Figure 2A middle panel, “Ye co-infection”), some Ye will enter locations near the epithelium. In the following paragraphs, we refer to these locations as the mucosal compartment, comprising the mucosa, the epithelium, and the gut-associated lymphatic tissues, such as the Peyer’s Patches (PP) and the overlying microfold cells (M-cells). Therefore, they elicit an innate immune response, with an increased AMP and increased cytokine production and infiltration of the professional phagocytes to the mucosal compartment [19,70]. For the sake of clarity, our model considers these factors not individually, but summarizes them into one abstract immune response. We assumed that this immune response affects, more strikingly, the endogenous microbiome and especially those commensals located at the mucosal compartment compared to Ye. In line with this, we observed a reduction in the diversity of the SI microbiome upon infection with Ye (Figure S1). The reduced complexity and density of the mucosa, near the microbiome, would then allow Ye to colonize and replicate there, if it is able to resist the host’s immune defense. Assuming a limited capacity of this mucosal compartment, all Ye exceeding the maximum capacity will drain into the lumen and finally end up in measurable CFUs in feces. Since only the Ye wt strain can cope adequately with the attack of phagocytes, both a YadA- and a T3SS-deficient strain will be quickly eliminated, despite the initial colonization, as experimentally observed by us (Figure 1) and others [19,28,29] (Figure 2A right panel; “after infection”).

The situation is very different in germ-free (GF) animals (Figure 2C,D). Here, no microbiota is present with which Ye would have to compete. Consequently, we assume that both the Ye wt and the mutant strain can expand within the gut lumen (Figure 2D). Additionally, the number of immune cells, M-cells, and the amount of AMP present in the mucus lining are presumably reduced, compared to SPF animals [71,72,73]. Thus, upon coinfection, the innate immune response to Ye is less intense compared to SPF-colonized animals (Figure 2D and Figure S2). Nonetheless, we expect that the Ye mutant strains may still be eliminated more efficiently compared to Ye wt at the mucosal compartment. This elimination might lead to a slow reduction of the mutant strain at late points in time after infection. Due to the lack of a microbiome in GF mice, we assume that both the Ye wt and the mutant strains will colonize the GIT at high numbers.

In the SPF-colonized *MyD88*^−/−^ mice that have a constricted immune response (Figure 2E,F), we also assume a weaker immune response. *MyD88*^−/−^ mice were reported to be highly susceptible to enteric infection [74,75,76,77]. Thus, we expect a faster progression of the infection. Moreover, we anticipate an amelioration of the difference between Ye wt and mutant strain CFUs during the infection course, because the better survival of Ye wt is primarily a result of its ability to cope with, and survive, the host’s immune reaction. As the immune system is only weakly active here, having the full capacity of the immune evasion mechanisms is no longer a clear advantage for the Ye wt strain. Thus, different outcomes of infection are conceivable (undecided, mutant wins, or wild-type wins); however, this depends on the total population size. As long as the total population size is reasonably large, one would expect equal levels of mutant and wild-type bacteria.

Additionally, we devised diagrams that depict our assumptions for the development of Ye CFU in feces for the strength of the host’s immune response (as assumed according to literature and some of our own qRT-PCR data; Figure S2). We performed a relative quantification of the mRNA levels of Reg3*γ*, Lipocalin-2, and S100A8 from mucosal scrapings as indicators of intestinal inflammation in SPF, GF, and *MyD88*^−/−^ mice that were coinfected with Ye wt and T3S0. Essentially, we found that in the SPF mice, all markers had higher basal levels compared to GF and *MyD88*^−/−^ mice, and that upon coinfection, all three markers of inflammation increased. An induction of S100A8 was also observed in the coinfected GF mice, but otherwise, the investigated markers were not significantly induced in the GF and *MyD88*^−/−^ mice. We are aware that this is a relatively shallow characterization of the host’s immune response at only a single point in time, but we think that, in the light of published data, it is justified to presume the immune response is most robust in SPF mice, less pronounced in GF mice, and weakest in the *MyD88*^−/−^ mice.

### 3.2. Mathematical Description of the Dynamic Population Model

#### 3.2.1. Presumptions Are Made for the Dynamic Population Model

**Figure 3:**
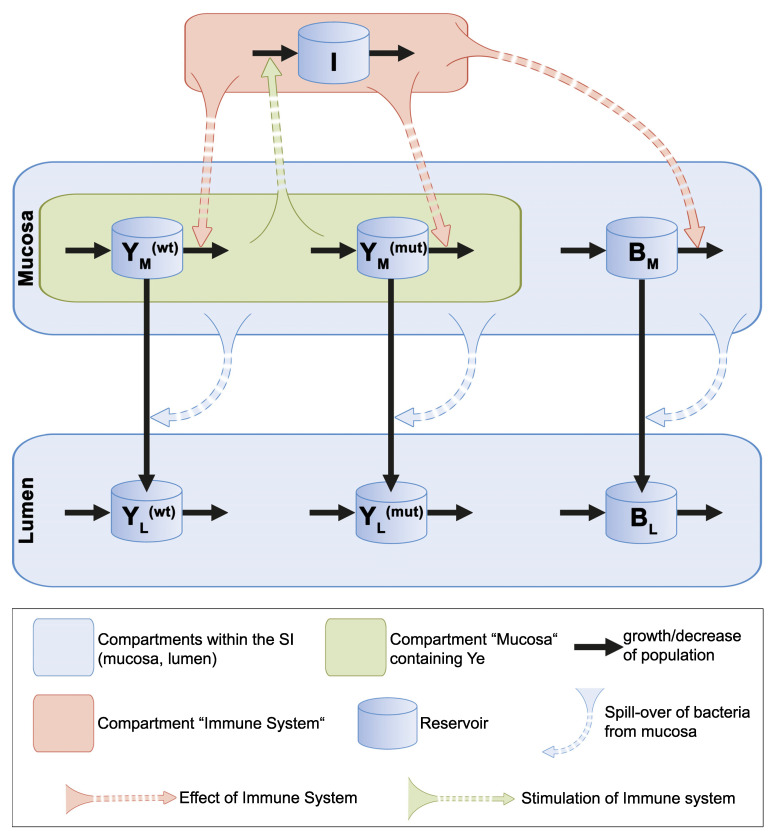
Schematic graphical depiction of the model composition and interaction networks. The model calculates population dynamics of the Ye wt (*Y_L_*^(*wt*)^; *Y_M_*^(*wt*)^) and mutant strains (*Y_L_*^(*mut*)^, *Y_M_*^(*mut*)^), as well as of commensal bacteria (*B_L_*; *B_M_*) at two different sites of the small intestine (SI), the luminal site and the extra-luminal mucosal site (“mucosa”; “lumen”). Additionally, it includes an abstract immune response with a distinct immune cell population (I). Bacterial and immune cell populations are illustrated as reservoirs. Individual growth rates determine the growth of bacterial populations. The decrease in populations is caused by intestinal peristaltic movement in the lumen and by immune killing in the mucosa. In addition, movement of bacteria from the mucosal compartment to the luminal compartment takes place. Upon entry of Ye wt or mutant strains to the mucosal compartment, they stimulate an immune response, which reciprocally affects all Ye and commensal populations within this compartment. The Ye wt strain, equipped with immune evasion factors, is less affected by the immune response than the Ye mutant strain, whereas both are more resistant than the commensal bacterial population (*B_M_*). Replicating populations that exceed the limited capacity of the mucosa drain into the lumen and, thereby, feed luminal populations. As a result of these bacterial population dynamics in the lumen, the model output is the calculated CFU of the bacteria ending up in feces. These curves are equivalent to experimental CFU data generated from the feces of orally infected mice.

We devised the most critical interactions among Ye, the host immune system, and the microbiota upon host entry and their impact on Ye population dynamics. We wanted these interactions to be included in the model and described them mathematically. The following presumptions for the computational modeling of *Yersinia* population dynamics were considered:

(1) The arrival in the SI and the colonization of a mucosal site: After an oral coinfection with a Ye wt and a mutant strain, both enter the lumen of the SI. A portion of these luminal Ye is then able to enter an extraluminal location, the mucosal compartment. If it is not attached to surfaces within the SI, bacteria will inevitably be transported towards the colon due to peristalsis. Within the colon, water will be reabsorbed from the intestinal content, and all bacteria finally end up in feces. Both the retention time and the replication rate of the bacteria determine how many bacteria will be detected in the feces at a distinct point in time. As Ye cells, presumably, have a lower replication rate than the endogenous microbiota, their CFU in feces would decline rapidly, compared to that of the commensals, if they do not establish a replicating population within the SI. However, experimental data show that the Ye CFU per g of content in the SI, at a later time of infection (7 dpi), is relatively high, especially in the distal part of the SI (Figure S3), and we have hints that there actually exists a niche within the GIT that can be colonized by Ye (Figure S4), see also the explanation under the following consideration, (2)). We hypothesize that Ye located in this mucosal compartment can resist its removal by peristalsis and can even replicate. Since this compartment would have a restricted capacity only, one basis of our model design is that all Ye cells exceeding this capacity will re-enter and feed the luminal populations and contribute to the CFU in feces.

(2) Bacterial interactions in the mucosal compartment: In our model, the mucosal compartment is considered a complex site which includes the mucosa, the epithelial lining, the lamina propria, and lymphatic tissues, such as the PP. Ye are known to adhere to mucus, degrade it, and invade the epithelium and PP [30,78,79]. Moreover, Ye expresses several virulence factors that facilitate an efficient immune evasion. This capability is especially important in the mucosal compartment, where the number of immune cells and the concentration of AMPs are high. Therefore, we assume that Ye can proliferate in the mucosal compartment, which is also colonized by a small number of commensal bacteria. The growth dynamics of both Ye and the commensal bacteria are determined by their initial numbers and their specific growth rates. Our model assumes that the whole endogenous microbiome has a higher growth rate compared to Ye (e.g., because the microbiome members are rather diverse and do not necessarily compete for nutrients or suitable niches). Notably, the combined number of all mucosal bacterial populations are restricted by a fixed capacity. Hence, Ye and members of the microbiota compete for the colonization of this compartment, and the further expansion of the population is only possible if the capacity limit has not been reached yet. Our own experimental data support our model hypothesis that an extra-luminal site is potentially feeding luminal Ye populations. We addressed this experimentally by orally coinfecting mice with Ye wt and Ye YadA0. Two days after infection, we assumed a successful colonization of the postulated niche (as seen before in comparable experimental settings). A systemic treatment with gentamicin (which does not permeate across the epithelial barrier) on 2 dpi considerably reduced Ye populations in the PP (Figure S4A). At the same time, Ye populations in feces were reduced (Figure S4B). As we could not observe a drop in total bacterial numbers at 3 dpi after the gentamicin treatment, we assumed that gentamicin did not enter the lumen (Figure S4C). Thus, we decided to integrate the abstract “mucosal compartment” as a Ye-specific niche into our model. However, the biological role of this niche remains to be elucidated.

(3) The influence of the immune system: Host immunity involves humoral and cellular factors. For the sake of simplicity, we summarized all host defense activities in one abstract immune action that only affects the mucosal compartment but is negligible in the luminal compartment. We hypothesize that the presence of Ye in the mucosal compartment activates the immune system. This activation increases proportionally to the number of Ye cells. The concentration of immune system effectors is highest close to the epithelium; therefore, we assume the immune system to influence the bacterial populations primarily at the mucosal site, compared to the bacteria within the lumen. As only the Ye wt strain has a full arsenal of virulence factors that allow for an efficient immune evasion, we assume that the Ye mutant strains and the commensal bacteria are killed more efficiently compared to the wild-type.

(4) Population dynamics and competition in the lumen: Most of the Ye applied orally during the initiation of the infection enter the luminal compartment that is already populated with microbiota. We assume the same bacterial growth rates in the luminal and mucosal compartments and set a limit to the total bacterial capacity of the lumen. It is known that within the SI, the bulk mass of bacteria resides in the lumen, whereas the host has evolved mechanisms to keep the majority of the microbiota distant from the epithelial surface lining. This bacterial distribution indicates that the capacity of the luminal compartment is conceivably larger than that of the mucosal compartment, which is also reflected by our model. The CFU of Ye in the luminal compartment over time is—as in the mucosal compartment—determined by the initial quantity of Ye and its distinct growth rate. Additionally, bacteria that exceed the capacity of the mucosal compartment spill over into the luminal compartment and, thereby, contribute to the CFU in the lumen. The most crucial difference between the two compartments in our model is that the immune reaction only affects the mucosal populations, and the luminal bacterial populations are reduced by the discharge of the intestine only. This choice neglects the role of, e.g., granulocytes in the intestinal lumen for the killing of Ye. However, we consciously decided to design the immune action this way because we wanted to keep the model manageable. We summarized and depicted all our considerations in Figure 3.

(5) The alignment of model output with experimental data: For technical reasons, the outputs of our wet-lab infection experiments are not the Ye CFU/g of content in the luminal compartment, but the CFU determined from feces after the colon passage, which has been concentrated by the reabsorption of water. Our model aims to simulate the population dynamics within the intestine. To be able to align our model output to our experimental data, we determined the mean percentage of the water content of different sections of the mouse gastrointestinal tract (Supplementary Table S1), carrying either SPF or GF microbiota, considering that the small intestinal content is massively thickened to be excreted as a solid fecal pellet. Based on these data, we have calculated a “thickening factor” that allows us to align our model output and the experimentally determined CFU values derived from fecal pellets (for a detailed description of the calculation, please refer to the Material and Methods section).

#### 3.2.2. Ordinary Differential Equations Describe the Dynamic Population Model

Based on the experimental data and theoretical considerations, we came up with the following mathematical model. As pointed out above, we assume that, following oral infection, a 1:1 mixture of the Ye wt and the mutant strains enter the SI. Most of the Ye remains in the lumen, but a small number enters the mucosal compartment. We assume that commensal bacteria already populate this location. The growth dynamics of the commensal bacteria *B_M_*, the wild type $Y_{M}^{\left(wt\right)}$, and the mutant strain $Y_{M}^{\left(mut\right)}$ in the mucosal compartment are determined by their quantities and by their growth rates, as described by a logistic growth with a maximum possible size. The growth rate *α*^(*B*)^ of the endogenous commensal bacteria is, presumably, higher than the Ye growth rates *α*^(*wt*)^ and *α*^(*mut*)^, respectively.

Moreover, the growth rates *α*^(*wt*)^ and *α*^(*mut*)^ are assumed to be equal. The capacity *C_M_* limits the expansion of the bacterial population in the mucosal compartment. When bacterial counts exceed this capacity, bacteria spill over to the lumen at the following rates σ:

$$\sigma_{M\to L\;}^{\left(wt\right)}=\alpha^{\left(wt\right)}\frac{B_{M}+Y_{M}^{\left(wt\right)}+Y_{M}^{\left(mut\right)}}{C_{M}},\;\sigma_{M\to L\;}^{\left(mut\right)}=\alpha^{\left(mut\right)}\frac{B_{M}+Y_{M}^{\left(wt\right)}+Y_{M}^{\left(mut\right)}}{C_{M}},\;\mathrm{and}\;\sigma_{M\to L\;}^{\left(B\right)}=\alpha^{\left(B\right)}\frac{B_{M}+Y_{M}^{\left(wt\right)}+Y_{M}^{\left(mut\right)}}{C_{M}}.$$

A variable that determines the infection course is the host’s immune system I. In the presence of Ye in the mucosa *Y_M_*, I is stimulated at rate κ but its strength is limited to a capacity *C_I_*, resulting in a logistic growth $\left(Y_{M}^{\left(wt\right)}+Y_{M}^{\left(mut\right)}\right)\cdot \kappa \cdot \frac{C_{I}-I}{C_{I}}$.

*I* directly acts on the bacteria present in the mucosa but indirectly influences the luminal populations by affecting the spillover from the mucosal compartment into the lumen. The immune system kills $Y_{M}^{\left(mut\right)}$ more efficiently than $Y_{M}^{\left(wt\right)}$, which has a full arsenal of virulence factors that allow for an efficient immune evasion. However, members of the commensal microbiome *B_M_* are the most susceptible to killing by *I*. This killing is modeled by using the term ($\gamma \cdot I\cdot B_{M})$, where γ is the immunity action rate. We use the adjustment factors $f_{\gamma }^{\left(wt\right)}$ and $f_{\gamma }^{\left(mut\right)}$ to account for the different susceptibilities of $Y_{M}^{\left(wt\right)}$ and $Y_{M}^{\left(mut\right)}$ towards killing by I and the even higher susceptibility of *B_M_*. The following differential equations describe the resulting dynamics of bacterial populations and immunity strength at the mucosal site:

(1) $$\frac{dY_{M}^{\left(wt\right)}}{dt}=\left(\alpha^{\left(wt\right)}-\sigma_{M\to L}^{\left(wt\right)}-\gamma \cdot f_{\gamma }^{\left(wt\right)}\cdot I\right)\cdot Y_{M}^{\left(wt\right)}$$

(2) $$\frac{dY_{M}^{\left(mut\right)}}{dt}=\left(\alpha^{\left(mut\right)}-\sigma_{M\to L}^{\left(mut\right)}-\gamma \cdot f_{\gamma }^{\left(mut\right)}\cdot I\right)\cdot Y_{M}^{\left(mut\right)}$$

(3) $$\frac{dB_{M}}{dt}=\left(\alpha^{\left(B\right)}-\sigma_{M\to L}^{\left(B\right)}-\gamma \cdot I\right)\cdot B_{M}$$

(4) $$\frac{dI}{dt}=\left(Y_{M}^{\left(wt\right)}+Y_{M}^{\left(mut\right)}\right)\cdot \kappa \cdot \frac{C_{I}-I}{C_{I}}$$

Most of the Ye from the oral infection enter the lumen of the SI. Additionally, luminal populations are fed by bacterial spillover from the mucosal compartment. The lumen is already populated with commensal bacteria. For the sake of simplicity, we use the same bacterial growth rates *α^(B)^*, *α^(wt)^*, and *α^(mut)^* in the lumen, as at the mucosal site. When we limit the total bacterial capacity of the lumen to a large number of *C_L_*, we obtain the following logistic growth for the luminal compartment:

$$\alpha_{L}^{\left(wt\right)}=\alpha^{\left(wt\right)}\frac{C_{L}-\left(B_{L}+Y_{L}^{\left(wt\right)}+Y_{L}^{\left(mut\right)}\right)}{C_{L}},\;\alpha_{L}^{\left(mut\right)}=\alpha^{\left(mut\right)}\frac{C_{L}-\left(B_{L}+Y_{L}^{\left(wt\right)}+Y_{L}^{\left(mut\right)}\right)}{C_{L}},\;\mathrm{and}\;\alpha_{L}^{\left(B\right)}=\alpha^{\left(B\right)}\frac{C_{L}-\left(B_{L}+Y_{L}^{\left(wt\right)}+Y_{L}^{\left(mut\right)}\right)}{C_{L}}.$$

Bacteria in the lumen move along the intestinal tract and are finally excreted at a removal rate *β*. Combining this, the following set of equations gives the dynamics of the bacterial populations in the lumen:

(5) $$\frac{dY_{L}^{\left(wt\right)}}{dt}=\left(\alpha_{L}^{\left(wt\right)}-\beta \right)\cdot Y_{L}^{\left(wt\right)}+\sigma_{M\to L}^{\left(wt\right)}\cdot Y_{M}^{\left(wt\right)}$$

(6) $$\frac{dY_{L}^{\left(mut\right)}}{dt}=\left(\alpha_{L}^{\left(mut\right)}-\beta \right)\cdot Y_{L}^{\left(mut\right)}+\sigma_{M\to L}^{\left(mut\right)}\cdot Y_{M}^{\left(mut\right)}$$

(7) $$\frac{dB_{L}}{dt}=\left(\alpha_{L}^{\left(B\right)}-\beta \right)\cdot B_{L}+\sigma_{M\to L}^{\left(B\right)}\cdot B_{M}$$

The resulting ordinary differential equation system, described in Equations (1)–(7), includes the dynamics of bacterial populations at the mucosal and luminal sites, as well as the immunity strength.

### 3.3. Validation of the Dynamic Population Model

#### 3.3.1. The Dynamic Population Model’s Parameters Were Estimated

To test the validity of the model and to see how well our computational model (Equations (1)–(7)) was defined, we estimated unknown parameter values in the ordinary differential equation system (Equations (1)–(7)) based on experimental data. We first aimed to reduce the number of parameters. This was achieved through experimental approaches, if possible, involving estimating biologically meaningful ranges for unknown parameters (based on the literature and our own data), or, at least, by defining the relations between distinct parameters (higher/lower/same). To this end, we experimentally determined the gut passage of time of C57BL/6J wild-type SPF (termed SPF from now on), C57BL/6J wild-type GF (termed GF from now on), and *MyD88^−/−^* SPF (termed *MyD88^−/−^* from now on) animals and found that in the GF animals, the gut passage of time was much longer than in SPF and *MyD88^−/−^* animals (Figure S5). We also determined the immunological parameters of SPF, GF, and *MyD88^−/−^* animals, thus supporting our assumptions regarding the relative strength of the immune response in the three particular systems (Figure S2).

To find reasonable values for parameters that either cannot be determined experimentally or can only be determined with a non-justifiable cost and effort, we started a computational parameter optimization to yield fits in the best agreement with the experimental data. Therefore, we used built-in optimization methods in MATLAB (see Materials and Methods). Detailed information for all parameters (such as definitions, the source of parameter values, functions, and relations to other parameters) is given in Table 1.

Of note, the model implementation and the optimization process were, at first, based on the dataset generated from the coinfection of SPF wild-type mice with the Ye wt and the YadA0 mutants.

#### 3.3.2. Parameters Were Fitted Based on the Coinfection Experiments in SPF Mice

**Figure 4:**
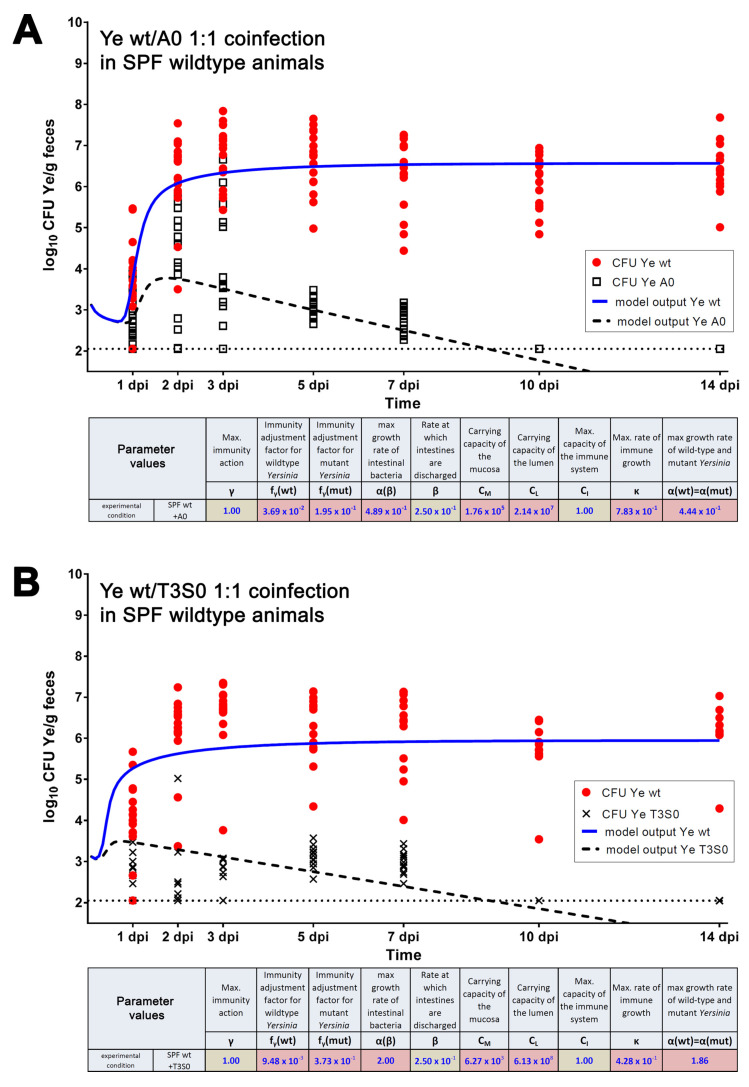
Overlay of model output and experimentally determined CFU values during Ye coinfection of SPF wild-type mice. When fitting the model to our experimental data, we obtained the parameter values listed in the inset tables. (**A**) Model output for CFU of Ye wt and Ye YadA0 shown as an overlay with experimental data. CFU values of individual animals at indicated points of time are shown for Ye wt and Ye YadA0. The dotted line indicates the limit of detection of our experimental system. (**B**) Model output for CFU of Ye wt and Ye T3S0 as an overlay with experimentally determined CFU values from the Ye wt:Ye T3S0 coinfection of SPF wild-type mice. The tables indicate fixed and calculated parameter values with green or red backgrounds, respectively; dpi = days post-infection.

We fitted our model to the Ye CFU data that were determined experimentally in coinfections of SPF mice with Ye wt and Ye YadA0 (Figure 4A). The independent estimation of parameters based on the second experimental dataset that was obtained by the coinfection of SPF mice with Ye wt and Ye T3S0 delivered slightly different, but comparable, absolute parameter values compared to Ye wt:Ye YadA0 coinfection. Hence, we observed a concordance of the model output with the experimental data (Figure 4B). Strikingly, the model even reflects a difference between the dynamics of CFU development of the Ye YadA0 and the Ye T3S0 strains; however, for the Ye wt:Ye T3S0 coinfection, the fit is relatively poor. This result has been predicted, since coinfection is one of the different mechanisms which occurs in multi-strain models. This mechanism is mathematically called the coexistence scenario, which refers to the simultaneous persistence of multiple infection populations with different life-history strains. In a further analysis of the differential equation model (Equations (1)–(7)), it was shown that a coexistence scenario could take place if the growth rates of the wt and mutant strains differed, even if this difference was subtle [80].

Moreover, our experimental finding, that Ye T3S0 is more susceptible to killing compared to Ye YadA0, is also corroborated by the model. Looking at the relative values of Ye wt ($f_{\gamma }^{\left(mut\right)}/\;f_{\gamma }^{\left(wt\right)})$, the Ye YadA0 strain is approximately 5 times more susceptible to killing by the immune system, whereas Ye T3S0 is approximately 40 times more susceptible, compared to Ye wt. The calculated parameter values obtained for these experimental datasets are depicted as insets in Figure 4.

Taken together, we were able to fit our experimental data and the model calculations for both coinfection settings in SPF mice. Therefore, we show that the model can reflect the processes taking place in the in vivo experiments. Despite the high number of free parameters, we obtained a proper fit.

#### 3.3.3. A Sensitivity Analysis of the Estimated Parameters Was Conducted

To better comprehend how sensitive the model is to changes in parameters, we analyzed the dynamic range of the model output by adopting different relations of the parameters $\;f_{\gamma }^{\left(wt\right)}$ and $f_{\gamma }^{\left(mut\right)}$ and checking the impact of these modulations on CFU development (Figure S6). This analysis revealed that the model is sensitive to changes within a range of relations of the parameters $\;f_{\gamma }^{\left(wt\right)}$ and $f_{\gamma }^{\left(mut\right)}$ of up to approximately 10, depending on the overall susceptibility of the individual mutant strains.

Essentially, models with estimated parameters can be validated in two ways: either the parameters are estimated for one dataset and are used to achieve the results from a second dataset, or the parameter values are estimated for two datasets and are, subsequently, compared. Therefore, we checked whether we could fit our experimental data for the Ye wt/YadA0 coinfection while using the parameter set obtained for the Ye wt/T3S0 coinfection, and vice versa (Figure S7; see Figure 4A,B for original values obtained for $f_{\gamma }^{\left(wt\right)},\;f_{\gamma }^{\left(mut\right)},\;\kappa$). Missing parameters were estimated by running an optimization with the parameter values for *γ*, *β*, and CI fixed, and using the experimental dataset of the Ye wt/YadA0 coinfection (Figure S7A). The same was done in reverse, using the wt/YadA0 parameter set and the wt/T3S0 experimental dataset (Figure S7B). We found that the parameter  κ (rate of immune growth), which adopted similar values in the independent estimations (7.83 × 10^−1^ for the Ye wt/YadA0, compared to 4.28 × 10^−1^ for the Ye wt/T3S0 setting; see Figure 4), was now widely diverged (8.61 × 10^−4^ for the Ye wt/YadA0 compared to 9.72 × 10^−1^ for the Ye wt/T3S0). In principle, the only differences between the wt/YadA0 and the wt/T3S0 coinfections were the different susceptibilities to killing by the immune system, compared to the wt. On the one hand, these validations corroborated our previous findings, with Ye T3S0 being most susceptible to killing by the immune system (approximately factor 25 compared to Ye wt) and Ye YadA0 being more susceptible compared to the wild-type (approximately factor 6), but much less susceptible compared to T3S0. However, our findings also mean that we can obtain a proper fit only when allowing all unknown parameters to run free. When using parameters derived from the other respective experimental settings, the model can calculate a CFU development roughly in line with the experimental data; however, it is with poor precision, as shown in Figure S7B. This underlines the strong relationship between the accuracy of parameter values and the quality of the model output and shows that the current model has clear limitations. It also demonstrates how the model can serve as a hypothesis generator that could be validated experimentally to refine the model. Further analyses on parameter sensitivity were conducted in [80].

### 3.4. Refinement of the Dynamic Population Model

#### 3.4.1. The First Model Refinement Was Based on Coinfection Experiments in GF Mice

**Figure 5:**
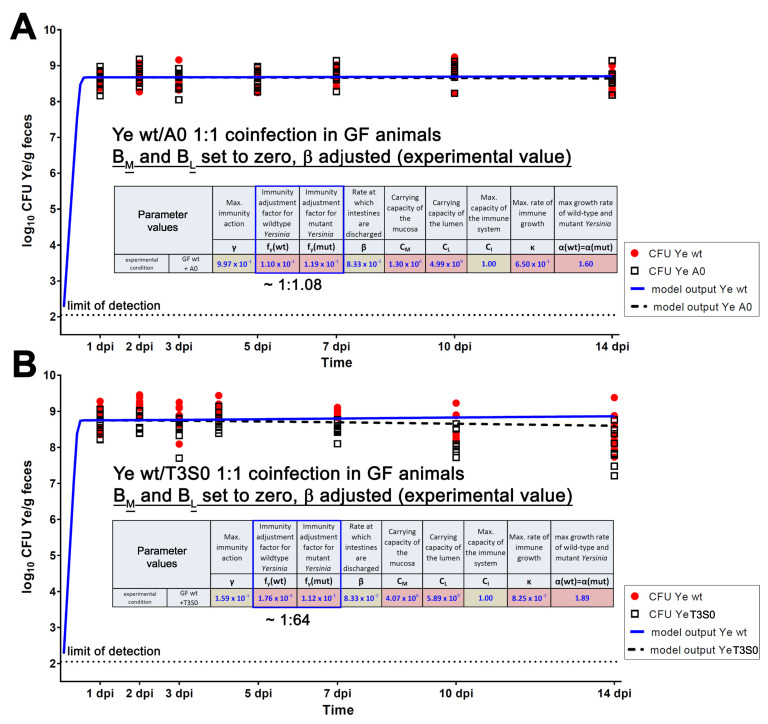
Infection course in the absence of microbiota. (**A**) Overlay of model output for CFU of Ye wt and Ye YadA0 or (**B**) Ye wt and Ye T3S0, and experimentally determined CFU levels from coinfections of GF mice. All parameters were estimated based on respective experimental data (parameter values are listed in the inset table); dpi = days post-infection.

To decipher how the model should be refined in different scenarios, we first generated a dataset using GF mice to mimic the lack of microbiota. Different basic parameter settings for microbiota-derived CR and host immune competence were adapted, and the resulting model calculations were analyzed by fitting them to experimental coinfection data. To decipher the effect of the absence of the microbiota on CFU development, we defined the number of *B_M_* and *B_L_* (i.e., number of bacteria in mucosal (M) and luminal compartment (L)) to be 0. Moreover, we considered that the fecal pellets have a higher water content in GF mice, as experimentally determined (Table S1). The higher water content was reflected by using a different thickening factor (0.2 instead of 1.3; for the calculation of the factor, please refer to the Materials and Methods section) to align the model output with the experimental data. Furthermore, we considered the lower discharge rate of intestines in GF mice (12 h mean residence time instead of 4.5 h in SPF animals), which we had also determined experimentally (Figure S5). The experimental coinfection of GF mice with Ye wt + Ye YadA0 or Ye wt + Ye T3S0, respectively, revealed that both the Ye wt and the mutant strains reached remarkably higher cell counts in feces compared to CFU levels in SPF-colonized mice. The T3S0 strain exhibited a slight attenuation, resulting in apparently lower CFUs, particularly from 7 dpi onwards, whereas Ye wt and Ye YadA0 counts remained constant at a high level over the entire observation period of 14 days (Figure 5). Our data, thus, indicates that in the absence of a commensal microbiome, both YadA and the T3SS seem to be dispensable for the effective colonization of the GIT.

We ran the model for the Ye wt/YadA0 coinfection setting only having defined boundaries for some parameters that were justified from a biological point of view (Table 1), as well as values we had determined experimentally. We obtained a proper fit of the model output and the experimentally determined course of CFU development (Figure 5A). The same was true for the Ye wt/T3S0 coinfection setting (Figure 5B). The most striking differences in parameter values, compared to the values we had obtained previously for the SPF wild-type model, were the higher capacities *C_M_* and *C_L_* for the mucosa and the lumen, respectively. This makes sense from a biological point of view, as GF animals have massively enlarged intestines. Interestingly, $\;f_{\gamma }^{\left(wt\right)}$ and $f_{\gamma }^{\left(mut\right)}$ were estimated to be very similar (0.110 for Ye wt and 0.119 for Ye YadA0). This finding might support our interpretation of the infection course in GF mice. Here, the Ye YadA0 strain does not have any disadvantage compared to the Ye wt strain and can expand within the gut to the same extent. Similarly, also in the model, YadA seems to be dispensable for colonization in the absence of a microbiota. By estimating the immunity action rate *γ* using this setting, we obtained an optimized value of approximately 0.997 (Figure 5A), which is very similar, compared to SPF. This finding was rather surprising as we had expected a lower activity of the immune system in the GF setting, according to the literature and our own data. However, our model calculates that the overall influence of *γ* on the expansion of Ye is only subtle (see Figure S8**,** where the CFU development of Ye wt and Ye YadA0 was calculated in the GF system while adopting values for *γ* between 1 (where the immune system is fully active) and 0 (no immune activity)). This can be explained by the absence of the endogenous microbiota that competes with Ye to fill the capacity of the small intestine in the SPF animals. We also modeled the GF Ye wt/T3S0 coinfection and obtained very similar results, compared to the Ye wt/YadA0 coinfection. The most apparent difference was that the fit for the T3S0 mutant strain CFU dropped slightly towards the end of our observation period, which is in line with our experimental data. Again, this difference in the behavior of Ye YadA0 and Ye T3S0 can be explained by their different susceptibilities to killing by the host’s immune system. In the absence of a microbiome, both strains can expand very quickly, and the effect of the enhanced killing of Ye T3S0 by the immune system is not as notable as in the SPF model system (Figure 5B). Taken together, we can obtain a fit where the model output is in alignment with our experimental data under GF conditions.

#### 3.4.2. An Immunocompromised Host Is Mimicked

The host’s immune system fundamentally influences the outcome and course of infection. Severe infections often occur when the function of the immune system is impaired. Thus, we wanted to test if we could get a proper fit for our experimental data and model output when simulating a host with impaired immune function. MyD88 is one of the key adaptor molecules involved in the activation of a sophisticated antimicrobial program that is initiated upon the binding of pathogen-associated molecular patterns to, e.g., toll-like receptors [81]. We made use of *MyD88*^−/−^ C57BL/6J mice that were colonized with a complex SPF microbiome as a model to decipher the role of a restricted immune response for Ye population dynamics. We assumed a more rapid and frequent invasion due to the reduction of the immune response, as depicted in Figure 2E,F (middle panel; “Ye coinfection”). As in the SPF wild-type model, Ye encounters the mucosal compartment occupied by commensals in the *MyD88^−/−^* animals. Because of the MyD88 deficiency, a much weaker immune response is induced. This, primarily, has two consequences: (i) The microbiota is less disturbed and is reduced. Therefore, Ye is less successful in establishing a population in the mucosal compartment, and the Ye counts will be lower. As the mucosal compartment feeds the luminal Ye population by its spillover, we will observe a lower Ye CFU in the GIT, compared to C57BL/6J wild-type animals. (ii) Due to the weak immune response of the *MyD88^−/−^* animals, we assume that the disadvantage of the mutant strains, in competition with Ye wt, is much less pronounced.

**Figure 6:**
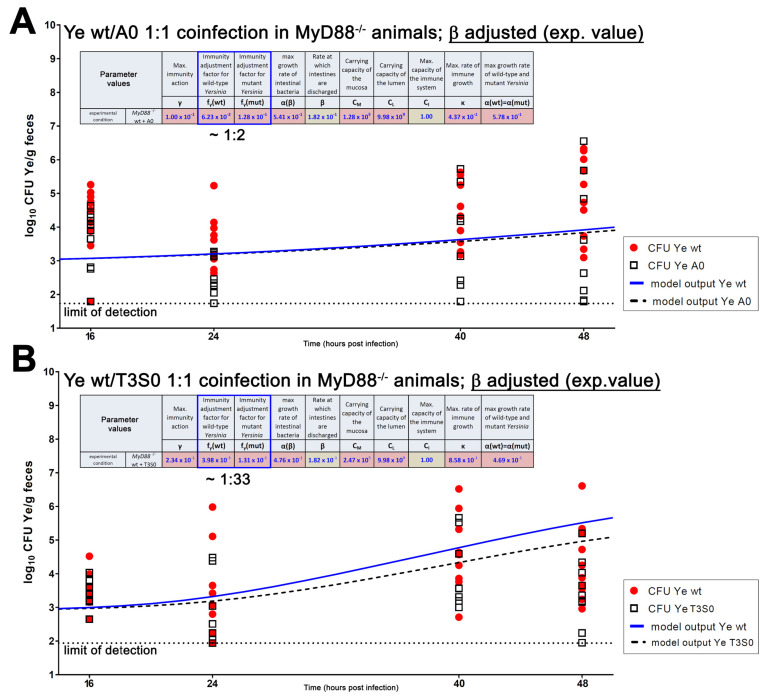
Infection course with an impaired immune response (*MyD88^−/−^*). (**A**) Overlay of model output and experimentally determined CFU levels from coinfections of SPF *MyD88^−/−^* mice with Ye wt and Ye YadA0 and (**B**) Ye wt and Ye T3S0. All parameters were estimated based on the respective experimental data (parameter values are listed in the inset table); dpi = days post-infection.

Finally, we coinfected SPF-colonized *MyD88^−/−^* mice, as described before. To compare the experimental results and modeling data, we created an overlay of the model output and the experimental data (Figure 6).

Due to the high frequency of systemic dissemination that has been observed with *Salmonella typhimurium* and *Citrobacter rodentium* [74,76,82], infections with Ye were conducted for two days only. To get a better temporal resolution within this shorter observation period, the Ye counts in feces were determined at two additional points of time (i.e., after 16 and 40 h). Within 48 h post-infection, the CFU of the Ye wt showed a slight increase compared to earlier points in time, but it never reached the high counts we observed in SPF wild-type mice. The mean CFU of the Ye YadA0 was marginally lower compared to that of Ye wt (Figure 6A), whereas the difference in CFU of Ye wt, compared to Ye T3S0, was more pronounced, but also subtle (Figure 6B). In some of the *MyD88*^−/−^ mice, the YadA0 and, to a lesser extent, the T3S0 strains reached a comparable, or higher, CFU, compared to the Ye wt strain, at 48 hpi. The stochastic detection of the mutants or wild-types are, presumably, the result of a very small total population size. As the CFU data are more scattered compared to the previous infection experiments, the fit is obviously less satisfying. This might be caused by the infection becoming systemic in some animals at these early points in time. However, we cannot control for this appropriately in this infection model. In summary, our experimental data show that, in the *MyD88^−/−^* SPF animals, a proper immune response outreaches the importance of the presence of the microbiome in preventing colonization and infection with Ye, and both YadA and the T3SS seem to play only a minor role in the colonization of the GIT. Again, we were able to fit our experimental data and the model calculations for both coinfection settings in *MyD88^−/−^* SPF mice. However, as the CFU data are relatively scattered due to the intrinsic properties of the mouse model, our fit is less reliable.

## 4. Discussion

The complex interplay of a specific pathogen with host factors, as well as the integrity and composition of the endogenous microbiome, determines the outcome of a gastrointestinal infection. Herein, we developed a mechanistic model and tried to fit original mouse infection data to it to identify differences between distinct experimental settings. By our attempts to rebuild the in vivo situation, we aimed to generate hypotheses that can explain our findings and can be validated experimentally in the future, to further improve the model design. Compared to other computational models of infection, the strengths of our model is the comprehensive experimental dataset underlying our study and its flexibility that can account for different host and pathogen properties. Based on experimental data obtained by oral mouse infections with Ye, we devised the specific entities and parameters that should be included in the model. Three distinct entities, with their particular population dynamics, were defined: a luminal compartment, a mucosal compartment, and the host’s immune response. Within these entities, the model considers the following aspects: (I) bacterial growth and release by fecal shedding, (II) the presence/absence of CR, mediated by the microbiome, (III) the role of specific virulence traits of the pathogen counteracting host immune factors, and (IV) the action of the immune system. The replication of all bacterial populations is the main contributor to population growth in both the luminal and the mucosal compartments. Populations exceeding the capacity of the mucosal compartment (whose capacity is assumed to be smaller than that of the luminal compartment) additionally feed the luminal populations. The distinct growth rates of the pathogen and the microbiota, as well as the capacities of the locations, were estimated. We assumed an overall higher growth rate of the complex microbiota, with its higher density and various requirements with regards to, e.g., the preferred nutrients and the oxygen availability. Its high diversity may reduce competition among the different phyla, as compared to the Ye populations that, presumably, have comparable requirements for optimal growth. Several approaches have been used in the past to unravel the growth dynamics of specific bacterial species within the gut microbiota [32,83,84,85]. Myhrvold et al. [83] determined that an *E. coli* strain, engineered for distributed cell division counting, had a doubling time of approximately three hours in orally-infected mice harboring a complex microbiota. The values we have estimated by our parameter optimization (*α*^(*wt*)^ = 0.44–1.89) were determined by in vitro cultures for the growth rates of Ye, which were in a comparable range to that of the mentioned *E. coli* strain. The growth rates we have estimated for the microbiota, as an entity, were surprisingly similar (*α*^(*B*)^ = 0.48–2.00); however, in almost all settings, they were slightly higher compared to that of the Ye strains. The absolute values we obtained for the growth rates of the Ye strains were moderately different between the Ye wt/YadA0 and Ye wt/T3S0 coinfection scenarios, e.g., in SPF animals, which aggravated an easy comparison of values. Therefore, we calculated the ratio of *α*^(*wt*)^/*α*^(*B*)^. This ratio turned out to be quite stable at approximately 0.9, which means that the endogenous microbiota only has a subtle growth advantage, compared to Ye, according to our model.

One weakness of our model is that it does not discriminate between the growth rates of the Ye wt and the mutant strains. Of course, we have determined their growth rates in in vitro liquid culture and have found them to be comparable, but we cannot rule out that they might behave differently within the host. Thus, one potent measure to enhance our model further would be to experimentally determine both the growth dynamics of Ye wt, YadA0, and T3S0 (in the different mouse models we used) and that of several representatives of the mouse gut microbiota. Alternatively, we could strive to implement already existing mathematical models that consider, not only the growth rate and the removal by fecal shedding, but also the death of the bacteria. Such an implemented model could also consider adaptation mechanisms that lead to a decrease in death, which are crucial for creating a steady state at later points in time after infection [83]. Another level of complexity could be reached by additionally considering interspecies competition and external perturbations [85], but this is beyond the scope of this study that was meant to be a starting point to establish more complex models in the future.

One crucial component of our model is the rather abstract “mucosal compartment”. We designed it in a way that the bacterial replication rate (per bacteria present) is constant within this compartment, and that the loss rate towards the lumen increases with the bacterial concentration in this compartment. There is some preliminary experimental evidence that Ye resides in such an extra-luminal site (cleaning niche experiment, Figure S4). However, it remains to be elucidated as to what this compartment looks like, how it can be occupied by Ye, which replication rate is adopted there, whether this rate remains stable over the entire observation period, and when Ye shed from this compartment into the lumen. This demands highly sophisticated experimentation and would be a project in itself, and is, therefore, beyond the focus of this work. Another possibility for the design of the mucosal compartment would have been, similar to the lumen, to assume a replication rate that decreases with an increasing bacterial concentration, and a fixed loss rate from this compartment to the lumen. However, for this design, experimental evidence would have to be generated.

The distinct entities of the mucosal and the luminal compartments exhibit different total capacities in nature and in our model. Rather than the exact values for these capacities, their relationship is of primary importance for our model. Natural barriers, such as the mucus layer and a high concentration of AMPs, limit the access of bacteria to the mucosal compartment [67,69]. Therefore, we implemented a considerably lower capacity of the mucosal site, compared to the lumen. The estimated overall capacity of the lumen is in good agreement with the Ye numbers that we determined in the feces of Ye co-colonized GF mice (where Ye can occupy the entire capacity, Figure 5), as well as with the numbers of cultivable commensal bacteria determined from different intestinal compartments (Figure S3). Myhrvold et al. [83] estimated, in their model, a similar carrying capacity of 10^9^ CFU/mL feces. Vaishnava et al. [86] assessed bacterial numbers in the murine mucosa and lumen of wt and *MyD88^−/−^* mice by quantitative PCR and the determination of the total 16S rRNA gene copy numbers. In wt mice, they detected considerably lower gene copy numbers in the mucosa compared to the lumen. Furthermore, FISH analyses of SI sections could show that secreted AMPs maintain a zone that efficiently eliminates bacteria close to the epithelium, and bacteria penetrate this barrier seldomly. In summary, these data support our model’s assumption of a capacity that is lower compared to that of the luminal compartment that is low in absolute numbers.

Intestinal peristalsis greatly influences the mean residence time of bacterial populations in the GIT and is causative for the dynamics with which bacteria end up in measurable counts in feces [10,83,87]. Our model takes this movement into account exclusively for the luminal compartment. We determined the mean residence time of particles in the GIT as a model parameter. To this end, we orally administered mice with fluorescent beads and monitored the excretion of beads over a time course of 24 h (Figure S5). In SPF-colonized wild-type mice, we defined a mean residence time of 4 h (3–5 h, *n* = 2), which was slightly different in *MyD88^−/−^* mice (mean 5.5 h, *n* = 2). These values are in fair agreement with data generated by other groups, who determined a transit time of approximately 6 h [83,87]. The mean residence time in GF mice was considerably higher than in SPF mice (12 h, *n* = 2). This can be explained by the enlarged cecum that lacks bacterial mucus degradation and has reduced peristalsis. Both are causative for a reduced defecation frequency and are well-known characteristics of GF animals [88]. Similar effects have been described for mice receiving long-term antibiotic treatment [89]. We are aware that the usage of GF mice has both its benefits and limitations. On the one hand, GF animals allow us to simulate a situation where the endogenous microbiome has been extinguished; however, it is without the side effects that may have been caused by antibiotic treatment (such as barrier defects). This allows a clear-cut interpretation of experimental data with regards to the role of the endogenous microbiome for the course of the infection. On the other hand, GF mice suffer from immunological defects, which limits the relevance of findings with regards to the role of the immune system [90]. Further optimization of the model could, thus, be achieved by performing infection experiments with animals that received broad-spectrum antibiotic treatment, or with mice harboring a defined microbiota [91,92,93].

The dynamics of the intestinal microbiota composition have previously been addressed in modeling approaches, especially in the context of a *Clostridium difficile* infection. Time-dependent metagenomics data were used to analyze the influence of antibiotic perturbations on microbiota and pathogen overgrowth in silico [85,94]. An adaption of this specific model, which combines a Lotka–Volterra model of population dynamics and regression, could lead to more elaborate model calculations, in terms of microbiota perturbations due to antibiotics, in the future.

In the current state of our model, the host’s immune response is implemented as one abstract parameter without a distinction between the actions of different cell populations of the innate and adaptive immune responses. We are aware that real life is much more complex. However, this integration of all immune system actions has the advantage of an easy adjustability of its activity that allows it to model, e.g., different peculiarities of immune deficiencies. In recent years, several mathematical models were developed to mirror bacterial gastrointestinal infection [32,34,35,95,96,97,98], viral infection at epithelial sites [38], and inflammatory disorders such as IBD [38,40]. Many of these studies had a clear focus on the host’s immune response, addressing the complex network that is activated by a given pathogen [38,40,96,99]. This was not the aim of this study, but future adaptations of the model could include a more sophisticated immune system and, thereby, could amplify the flexibility of the model.

In our model setup, the immune response is stimulated by the entry of Ye into the mucosal compartment. The strength of the immune action correlates with the numbers of Ye present at this site. All bacterial populations at the mucosal site are affected by the stimulated immune response, but the model can account for the different immune evasion potentials of the infecting pathogen. The capacities to evade the immune system can be adjusted individually by varying the immunity adjustment factors *f_γ_*. In contrast to the approaches mentioned earlier, our model allows the simultaneous and independent modulation of virulence, the growth rate of the pathogen, and the underlying host conditions with respect to immune competence and CR. By modulating relevant host conditions (the presence of microbiota and the functionality of the immune system), we finally tested whether our model could reflect these profound changes. We obtained a good fit of our data from the infections of SPF mice. The fit even reflected the difference between the two mutant strains of Ye towards its killing by the immune system. However, one crucial issue that needed to be resolved to carry out correct parameter optimization was to figure out how to handle CFU values that were at, or below, our detection limit. This problem could be resolved mathematically (please refer to the Materials and Methods section for details).

In our study, we adopted an experimental scenario where the endogenous microbiota was utterly lacking. To our surprise, both the Ye wt and the mutant strains were able to reach very high CFU levels, filling up the entire available capacity of the intestines. For initial model calculations, we used parameters derived from the Ye wt/YadA0 coinfection, but we considered a longer passage time, a higher water content of the feces of germ-free mice, and of course, the absence of microbiota, while assuming the immune system was as active as in the SPF model. We know that the immune system of GF animals is not as developed as in SPF wild-type animals (see Figure S2; [90]), but further attempts to obtain a good fit of our data revealed that the activity of the immune system had only a minor impact on the CFU development in the absence of a microbiome (Figure S8). In sum, the model also reflected the course of infection in the GF system.

Next, we adopted a scenario with a limited immune response, experimentally mimicked by the usage of MyD88-deficient mice. Again, we obtained a fit of our experimental data; however, this was only within a short timeframe (48 h of infection, in line with the shortened experimental infection setting). As we could only model compartments within the GIT, we could not employ it when the infection becomes systemic, where the pathogen enters new compartments that are not included. The systemic dissemination of Ye presumably happens very quickly in *MyD88^−/−^* mice due to the compromised epithelial barrier functions [83,86]. We conclude that colonization of the PP is not necessarily a prerequisite for systemic spread. In fact, we even observed a lower abundance of Ye wt CFU in the feces of *MyD88^−/−^* mice, compared to an infection in immunocompetent SPF wild-type mice. We assume that the largely restricted immune response in the *MyD88^−/−^* mice is not sufficient to considerably decimate the commensal population during Ye infection. Therefore, the Ye population cannot expand as much as in the SPF animals, resulting in lower CFU amounts in the feces of the *MyD88^−/−^* mice, compared to the SPF mice. However, this needs experimental proof. To further elucidate the colonization of the mucosal compartment and dissemination events, as well as cellular immune responses, a histological approach is needed. We aimed to quantify how many Ye enter the Peyer’s Patches by sampling the PPs of infected mice at a point in time where the CFU was at its maximum value. We then generated serial sections and performed immunohistology; however, even with the very high CFUs obtained in feces during the infection course, this approach was not sensitive enough to detect Ye in the tissue sections. The extent to which the immune response affects commensal species in *MyD88^−/−^* animals, in their number and composition, could be addressed by a sequencing approach, as conducted for the SPF wild-type animals. However, this question was beyond the scope of this study.

Another feature that would greatly enhance the power of our model was developed by Miller et al. [100]. They implemented a multi-compartment model of symptomatic bacteremia. This could possibly be connected to our model of the GIT if the translocation rates of bacteria from the gut, into the bloodstream and other organs, could be determined. These authors also included the possibility to model the impact of antibiotic treatment on CFU development.

Our main findings are that the model can reflect the infection course in different host settings (an immune-competent host with a diverse microbiota, no microbiota, or one that is immunocompromised), with the caveat that we allowed for many parameters to adopt any value within a predefined range. Still, we found that similar parameters were obtained. However, each setting involves its own distinct parameter set to obtain the best fit. To calculate the CFUs during the infection course reliably, it was not enough to alter individual parameters to adopt a change implied by a specific condition (e.g., no microbiota present). This could only occur if parameter values were optimized based on the respective experimental dataset where the curve fits were in good agreement with our experimental observations. The model can now be improved with further model analyses and enhancements, based on our findings. The differences in structural and functional details (e.g., GIT morphology, physiology, and gut passage of time), even in our basic experimental setting (comparing SPF and GF animals), presumably show that the parameter values are not merely exchangeable between systems. Within a consistent host condition and pathogen phenotype, however, the infection course should, in principle, be determined mathematically. A crucial step towards more reliable calculations would therefore be the reduction of unknown parameters.

## 5. Conclusions

We conclude, from our study, that to create a reasonable data-driven mechanistic model, an excellent understanding of the causative agent of GIT infection is needed: How does the pathogen interact with the host? Does it produce specific virulence factors? How do these factors contribute to population dynamics (e.g., by mediating an immune evasion)? Does the pathogen have specific requirements for growth (e.g., oxygen and nutrients)? These, and many more, questions need to be answered, or the corresponding parameters need to be clarified experimentally for as many parameters as possible. Consequently, our current model needs further refinement. More parameters need to be determined experimentally. To adapt the model to other pathogens, it would be necessary to implement changes that more precisely reflect the pathogen’s specific peculiarities with regards to the above-mentioned characteristics. Such adaptations might be implemented more quickly with pathogens that have a lifestyle comparable to that of Ye, but this will require profound changes of the model setup for other pathogens.

A good understanding of the infected host is also needed to create a model that delivers reasonable calculations: Is its microbiota able to mediate full colonization resistance? Was the microbiota already disturbed by medication? Is the immune system fully operable? Is the GIT physiology disturbed (leading to, e.g., prolonged or impeded gut passage)? The more detailed our understanding of the pathogen and the host, the better the model can reflect biology.

In sum, we think that computational modeling of infection has great potential, but also many caveats, such as the vast complexity of biological systems even under laboratory conditions and the plasticity of the causative pathogens. Importantly, computational modeling requires the close cooperation of disciplines that receive profoundly different training. For us, this was not trivial, and, therefore, we strongly support the suggestions by Vlazaki et al. [101] to implement interdisciplinary training of young academics who can exploit the potential of data-driven computational models.

## Figures and Tables

**Table 1:** Overview of all parameters and variables within the model. This table lists all sources of values, functions, relations to other parameters, and preset boundaries, as well as the exact values used for parameter calculation and the assumptions that we made to justify the choice of relations/preset boundaries.

Parameter	Definition	Source of Parameter Value	Function	Relation to Other/Comment	Preset Boundary/Exact Value	Assumptions Made to Justify the Choice of Preset Boundaries
Growth						
*α^(B)^*	Growth rate of commensal bacteria	Estimated	Adjustable growth rate of commensal bacteria	Higher compared to growth rate of Ye	0.4–2.0	High diversity and different requirements for growth enable overall faster growth compared to Ye.
*α^(wt)^*	Growth rate of the Ye wt	Estimated	Adjustable growth rate of the Ye wt strain	Same as growth rate *α^(mut)^*	0.4–2.0	Growth optimum of Ye is at 30 °C; all Ye have the same requirements and compete for nutrients. Therefore, they grow slower compared to the microbiota.
*α^(mut)^*	Growth rate of the Ye mutant strains	Estimated	By adjustment of the Ye mutant growth rate, the model can account for growth deficiencies.	Same as growth rate *α^(wt)^*	0.4–2.0	Mutant Ye do not have a growth defect, they just lack a virulence factor dispensable for normal growth; in vitro growth did not reveal a difference in the growth rate of wt and mutant Ye.
Discharge						
*β^(SPF)^*	Discharge rate of intestines	Experimental data (0.22/h)	Adjustable rate accounting for varying GIT passage times in different host models.	Higher as in *MyD88*^−/−^ and GF	0.22	Justified by experimental data.
*β^(GF)^*	Discharge rate of intestines	Experimental data (0.08/h)	Adjustable rate accounting for varying GIT passage times in different host models	Lower than in SPF and *MyD88*^−/−^	0.08	Justified by experimental data.
*β^(MyD88−/−)^*	Discharge rate of intestines	Experimental data (0.18/h)	Adjustable rate accounting for varying GIT passage times in different host models	Lower than in SPF, but higher compared to GF animals	0.18	Justified by experimental data.
Immunity action related						
*γ*	Immunity action rate	Adjustment factor for the immune action; 1 means 100% activity	Allows adjustment of the global immune action to account for immune deficiencies in a specific host.	Lower in GF and *MyD88*^−/−^	0.1–1.0	It is known that GF animals have a less developed immune system. *MyD88*^−/−^ animals suffer from reduced activity of the immune system (see Introduction for references).
*κ*	Rate of immune growth	Estimated	Allows adjusting the rate at which the immune response is activated.	Unknown	0.004–0.1	No justification.
*fγ^(wt)^*	Immunity adjustment factor of the Ye wt	Estimated	Allows adjustment of resistance of the Ye wt strain to immune killing and thereby accounts for immune evasion mechanisms of a pathogen.	Lowest compared to *fγ^(YadA0)^* and *fγ^(T3S0)^*	0.001–0.11	The Ye wt strain is most resistant to killing by the immune system due to its ability to evade the host immune system, e.g., by engaging its T3SS, or by recruiting negative regulators of complement by YadA (see Introduction for references).
*fγ^(YadA0)^*	Immunity adjustment factor of the Ye YadA0 strain	Estimated	Adjustment allows accounting for an increased (or reduced) susceptibility to immune killing due to mutations affecting Ye immune evasion mechanisms.	Higher compared to *fγ^(wt)^* but lower or equal in comparison to *fγ^(T3S0)^*	0.11–0.2	Ye YadA0 is less resistant to killing by the immune system compared to Ye wt.
*fγ^(T3S0)^*	Immunity adjustment factor of the Ye T3S0 strain	Estimated	Adjustment allows accounting for an increased (or reduced) susceptibility to immune killing due to mutations affecting Ye immune evasion mechanisms.	Higher compared to *fγ^(wt)^* and higher or equal compared to *fγ^(YadA0)^*	0.11–0.2	Ye T3S0 is less resistant to killing by the immune system compared to Ye wt and less resistant compared to Ye YadA0.
Compartment capacities						
*C_I_*	Capacity of the immune response	Predefined	Caps the maximum activity of the immune system.	*C_I_* = 1 means that the immune system is fully operative	≤1	Not applicable.
*C_M_*	Capacity of the mucosal site	Estimated	Caps the replication of the populations within the mucosa to an adjustable maximum capacity.	Lower than *C_L_*	10^3^–10^7^	Assumed range of commensal bacteria in proximity to the epithelium based on literature [73].
*C_L_*	Capacity of the luminal site	Estimated	Caps the replication of populations within the intestinal lumen to an adjustable maximum capacity.	Higher than *C_M_*	10^6^–10^10^	The total number of commensal bacteria in the distal small intestine is ~10^7^–10^10^ per mL.
Alignment of experimental data with model output						
Thickening factor	Reflects water extraction from fecal material during the colon passage	Experimental data	Allows adjusting experimentally measured CFU in fecal pellets and model-calculated CFU (within intestines).	-	SPF (1.3); *MyD88^−/−^*(1.3); GF (0.2)	Justified by experimental data

## Data Availability

The model is openly available in SBML format [102,103] from the BioModels Database [104] under accession number MODEL2002070001. 16S rRNA sequencing data will be published at the European Nucleotide Archive database with the accession number PRJEB50711.

## Supplementary Materials

The following supporting information can be downloaded at: https://www.mdpi.com/article/10.3390/biology11020297/s1, Figure S1: Impact of Ye infection on SI microbiome composition, Figure S2: Relative quantification of mRNA levels of Reg3*γ*, Lipocalin-2 and S100A8 from mucosal scrapings as indicators of intestinal inflammation, Figure S3: Distribution of Ye and cultivable commensals along the GIT, Figure S4: Systemic administration of gentamicin for cleansing of a potential extra-luminal niche colonized by Ye, Figure S5: Quantification of mean bacterial residence times in SPF-colonized or GF C57BL/6J wild-type mice and SPF-colonized *MyD88*^−/−^ animals, Figure S6: Dynamics of model output when adopting different relations between *f_Y_^(mut)^* and *f_Y_^(wt^*^)^, Figure S7: Mutual exchange of parameter values estimated for the wt/A0 and T3S0 setting and estimation of *f_Y_^(wt)^*, *f_Y_^(mut)^* and κ, Figure S8: Dynamics of model output in the GF infection setting when adopting different activities of the host immune system, Table S1: Mean percentage ± SD of water content in sections of the mouse GIT, Table S2: Strains and plasmids used in this study, Table S3: Oligonucleotides used in this study, Table S4: qRTPCR raw data, Table S5: Data set used to calibrate the model. File S1: Computational model in SBML format. File S2: Matlab script for parameter estimation.
