# Supplementary material for: A Computational Model of Bacterial Population Dynamics in Gastrointestinal Yersinia enterocolitica Infections in Mice
Source: Biology (Basel). 2022 Feb 12;11(2):297. doi: 10.3390/biology11020297 (PMC8869254; doi:10.3390/biology11020297)
Supplement: Supplementary file 1 [file biology-11-00297-s001.zip › Supplementary Captions.pdf]

Figure S1. Impact of Ye infection on SI microbiome composition. Microbial composition of SI contents of mice with different infection outcomes, as assessed by 16S rRNA sequencing. **(A)** Relative abundances of microbiota representatives on the family level. Samples were isolated from the SI after oral Ye wt infection. Relative abundances of families are shown in stacked bar charts for individual animals. Data were grouped according to the observed change of body weight at earlier time points (weight loss after 3-7 days (red), no weight loss between 1 dpi and 7 dpi (orange), or uninfected control group (blue). Long-term infected mice returned to a kind of a steady-state at a late time point of infection and had no signs of sickness anymore (yellow). **(B)** Taxonomic tree on the phylum-, class-, order- and family-level allowing the assignment of the color code used in (A). **(C)** Principal component analysis (PC) on Bray-Curtis dissimilarities of the microbial composition of samples. Color code reflects assignments to groups as in (A). **(D)** Impact of Ye infection on microbial diversity. Shannon diversity of the SI microbiome composition in the different groups of animals. Statistically significant differences were identified using an unpaired Wilcoxon sum rank test. ** $P < 0.001$.

Figure S2. Relative quantification of mRNA levels of Reg3γ, Lipocalin-2 and S100A8 from mucosal scrapings as indicators of intestinal inflammation. Relative expression levels compared to the housekeeping gene beta-glucuronidase were determined by qRT-PCR in mock-infected and mice co-infected for two days with Ye wt/Ye T3S0. **(A)** Basal expression levels and expression levels of Reg3γ following infection of SPF wild type mice, GF animals, and SPF-colonized *MyD88*$^{-/-}$ mice. **(B)** Expression levels of Lipocalin-2 **(C)** Expression levels of S100A8. A nonparametric Mann-Whitney test determined statistically significant differences between groups. ** $P < 0.01$.

Figure S3. Distribution of Ye and cultivable commensals along the GIT. At 7 dpi after oral infection of SPF-colonized C57BL/6J mice with the Ye wt strain, the numbers of Ye and cultivable commensals were determined in different compartments of the GIT. The small intestine was dissected and cut into three pieces of equal length (SI1, SI2, SI3). Additionally, the caecum and the colon were dissected. CFUs per gram of fecal content of the three SI pieces and of the caecum and the colon were determined by plating.

Figure S4. Systemic administration of gentamicin for cleansing of a potential extra-luminal niche colonized by Ye. Mice were orally co-infected with a 1:1 mixture of Ye wt and Ye YadA0 for two days. At this time point, the successful colonization of a potential niche was assumed. One group was then administered intraperitoneally with gentamicin and a control group with saline only. (**A**) Ye wt and Ye YadA0 CFU was determined from Peyer's patches (PP) on 3 dpi. (**B**) Ye CFU of both strains in feces on days 2 and 3 post-infection. (**C**) The impact of systemic gentamicin treatment on the total CFU at 3 dpi of all cultivable bacteria was addressed by the plating of feces on non-selective agar plates. A paired t-test assessed a statistically significant difference within the Gentamicin treatment group. $P = 0.494$.

Figure S5. Quantification of mean bacterial residence times in SPF-colonized or GF C57BL/6J wild type mice and SPF-colonized *MyD88*$^{-/-}$ animals. Two mice per group were orally challenged with $1 \cdot 10^9$ fluorescent polystyrene beads plus $5 \cdot 10^8$ CFU of Ye wt, and feces were collected hourly over 24 h. The number of fluorescent events/g feces at each time point was analyzed by flow cytometry. The cumulated bead-hours were calculated as shown in the heat maps, and the graphs are plotting the $\log_{10}$ of cumulated bead-hours for the individual animals. The mean residence time per bead was calculated by dividing the sum of events/g of feces through the total number of bead-hours.

Figure S6. Dynamics of model output when adopting different relations between $f_Y^{(mut)}$ and $f_Y^{(wt)}$. To visualize the impact of the relative susceptibility to killing by the immune system on population dynamics of the Ye YadA0 (left column) and the Ye T3S0 strain (right column) we plotted curves for

$f_Y^{(mut)}$ adopting values **(A)** equal to that of $f_Y^{(wt)}$, **(B)** 2x $f_Y^{(wt)}$, **(C)** 5x $f_Y^{(wt)}$, **(D)** 10x $f_Y^{(wt)}$, **(E)** 20x $f_Y^{(wt)}$and **(F)** 40x $f_Y^{(wt)}$ for the respective settings. The relationships that the best matched what our model calculated based on the experimental data are highlighted with a blue frame.

Figure S7. Mutual exchange of parameter values estimated for the wt/A0 and T3S0 setting and estimation of $f_Y^{(wt)}$, $f_Y^{(mut)}$ and κ. (A) Parameter values calculated for the Ye wt : Ye YadA0 coinfection were used to estimate $f_Y^{(wt)}$, $f_Y^{(mut)}$, and κ for the A0 experimental dataset and **(B)** vice versa. Model output for CFU of Ye wt and Ye A0/T3S0 using the newly estimated values of $f_Y^{(wt)}$, $f_Y^{(mut)}$, and κ as overlay with experimentally determined CFU values of infected SPF wild type mice. Starting values used for parameter estimation, parameters used for solving the system and the newly estimated value for $f_Y^{(wt)}$, $f_Y^{(mut)}$, and κ (highlighted) are indicated. Calculated parameter values (red background), and fixed parameter values (green background) are shown in the tables.

Figure S8. Dynamics of model output in the GF infection setting when adopting different activities of the host immune system. (A) Using the same experimental data and parameter set as in Fig. 5A, we calculated the CFU development after the coinfection of GF mice with Ye wt and Ye YadA0 with γ adopting values between 1 (immune system fully active) and 0 (no immune activity). **(B)** For better discrimination of curves, the scale of the y-axis was altered. Curves of the same color and pattern represent one dataset showing the CFU development for Ye wt in the upper half and that of Ye A0 in the lower half of the graph for a value of γ as indicated on the right side. High activity of the immune system correlates with more considerable expansion of the Ye wt strain and decrease of CFU of the Ye A0 strain, but the overall effect of changes in γ is subtle.

Table S1 Mean percentage ± SD of water content in sections of the mouse GIT. SI1, SI2, SI3 indicate the respective part of the SI that was analyzed. Please also refer to Figure S3.

Table S2 Strains and plasmids used in this study

Table S3 Oligonucleotides used in this study

Table S4 qRTPCR raw data

Table S5 The data set used to calibrate the model.
