# Supplementary material for: A Computational Model of Bacterial Population Dynamics in Gastrointestinal Yersinia enterocolitica Infections in Mice
Source: Biology (Basel). 2022 Feb 12;11(2):297. doi: 10.3390/biology11020297 (PMC8869254; doi:10.3390/biology11020297)
Supplement: Supplementary file 1 [file biology-11-00297-s001.zip › Table S1 water content.pdf]

Table S1. Mean percentage ± SD of water content in sections of the mouse GIT. SI1, SI2, SI3 indicate the respective part of the SI that was analyzed. Please also refer to Figure S3.

	**SI1 + ½ SI2**	**½ SI2 + SI3**	**Caecum**	**Colon**	**Fecal pellet**
**SPF**	74,64 ± 3,31	76,59 ± 3,85	75,42 ± 0,35	50,69 ± 16,31	29,66 ± 2,37
**GF**	73,11 ± 3,14	73,63 ± 0,63	78,76 ± 1,79	70,07 ± 1,07	49,01 ± 3,69
