# Supplementary material for: A Computational Model of Bacterial Population Dynamics in Gastrointestinal Yersinia enterocolitica Infections in Mice
Source: Biology (Basel). 2022 Feb 12;11(2):297. doi: 10.3390/biology11020297 (PMC8869254; doi:10.3390/biology11020297)
Supplement: Supplementary file 1 [file biology-11-00297-s001.zip › Table S2 Strains and plasmids used in this study.pdf]

Table S2 Strains and plasmids used in this study

Strain	Relevant characteristics	Resistance	Source
Ye WA-C	*Yersinia enterocolitica* WA-314 serotype O:8, lacking the pYV plasmid	Nal	(Heesemann 1987)
YadA wt	*Yersinia enterocolitica* WA-314 serotype O:8 with pYV plasmid YadA wt	Nal, Kan, Spec	(Schütz, Weiss et al. 2010)
YadA0	YadA deficient mutant of WA-314, generated by insertion of a kanamycin cassette	Nal, Kan	(Roggenkamp, Neuberger et al. 1995)
T3S0	pYV515 mutant strain of WA-314, deficient in Yop secretion, generated by Tn5 insertional inactivation of lcrD	Nal, Kan	(Ruckdeschel, Roggenkamp et al. 1996)
YadA0 CmR	YadA0 with chromosomal insertion of a chloramphenicol cassette into the YenI locus	Nal, Kan, Cm	this work
T3S0 CmR	T3S0 with chromosomal insertion of a chloramphenicol cassette in YenI locus	Nal, Kan, Cm	this work
E. coli 118λ pir	variant of *E. coli* CC 118, used to maintain suicide plasmids		(Herrero, de Lorenzo et al. 1990)
E. coli β2163	strain, carrying a Δdap::(erm-pir) and RP4 from *E. coli* SM10 used for conjugative transfer and counter selection	Kan	(Demarre, Guérout et al. 2005)
**Plasmid**	**Relevant characteristics**		**Source**
pSB890Y	suicide cloning vector with PstI restriction sites mutated	Tet	this work
pASK-IBA4C	expression plasmid with chloramphenicol^R cassette	Cm	IBA Lifesciences

Cm: Chloramphenicol; Kan: Kanamycin; Nal: Nalidixic acid; Spec: Spectinomycin; Tet: Tetracyclin
