# Supplementary material for: A Computational Model of Bacterial Population Dynamics in Gastrointestinal Yersinia enterocolitica Infections in Mice
Source: Biology (Basel). 2022 Feb 12;11(2):297. doi: 10.3390/biology11020297 (PMC8869254; doi:10.3390/biology11020297)
Supplement: Supplementary file 1 [file biology-11-00297-s001.zip › Table S3 Oligonucleotides used in this study.pdf]

Table S3 Oligonucleotides used in this study

Name	Sequence (5´-> 3´)	Description
**<u>Generation of mutants</u>**		
**gib_uni_890_r2**	CAAGAGGGTCATTATATTTCGCG	reverse (rev.) primer for linearization of pSB890Y
**gib_uni_890_f2**	CAAGCTCAATAAAAAGCCCCAC	forward (fwd.) primer for linearization of pSB890Y
**gib_Yen_up_f**	GTTATTCCGCGAAATATAATGACCCTCTTGCAGTACTTTCGCCCAAGAGC	fwd. primer for upstream fragment complementary to YenI locus with overlaps to PSB890Y
**gib_Yen_up r2**	GTGAAAGTTGGAACCTCTTACGTGCCGATCTGTCAATTCACTACCTCAGATC	rev. mutagenesis primer for upstream fragment complementary to YenI locus with overlaps to Cm cassette
**gib_Yen_camp_f2**	AATGGGATGATCTGAGGTAGTGAATTGACAGATCGGCACGTAAGAGGTTCC	fwd. primer for Cm gene in pASK-IBA4C with overlaps in YenI upstream fragment
**gib_Yen_camp_r2**	CTTTAAGGTTATCCATCAGAATGATTAATTCAAACCACCGCTGGTAGCGG	rev. primer for Cm gene in pASK-IBA4C with overlaps in YenI downstream fragment
**gib_Yen_down f2**	AAAAAAACCACCGCTACCAGCGGTGGTTTGAATTAATCATTCTGATGGATAACC	fwd. primer for downstream fragment complementary to YenI locus with overlaps to Cm cassette
**gib_Yen_down_r**	CCACCGCGGTGGGGCTTTTTATTGAGCTTGTTATGCTCGCCAAATTTTCC	rev. primer for downstream fragment complementary to YenI locus with overlaps to pSB890Y

**<u>Verification of mutations</u>**		
**p890_seq_f**	CGTCACCAAATGATGTTATTCC	fwd. primer for verification of constructs in pSB890Y
**p890_seq_r**	GTTGAGAAGCGGTGTAAGTG	rev. primer for verification of constructs in pSB890Y
**Yen_851_f**	TGGGTGAGATGGTGTTAGGC	sequencing primer for verification of construct in YenI locus
**Yen_1336_f**	CAGCTGGATATTACGGCCTTT	sequencing primer for verification of construct in YenI locus
**Yen_1849_f**	CAACAGTACTGCGATGAGTGG	sequencing primer for verification of construct in YenI locus
**Yen_2356_f**	CGCTAAAGAAGAAAGGGAAACA	sequencing primer for verification of construct in YenI locus
**Yen_2829_f**	CCCCTAATTTCCTCCCACTT	sequencing primer for verification of construct in YenI locus

**Yen_3200_r**	TTGATCTCTATTCCTGCATTTTT	sequencing primer for verification of construct in YenI locus
**Yen_3687_r**	TCGGTATGTACTGTCATCAATGTTT	sequencing primer for verification of construct in YenI locus
**<u>TaqMan Assays used for qRT-PCRs</u>**		
**assay**	**target**	**source**
**Mm00441127_m1**	Reg3γ	ThermoFisher Scientific
**Mm01197698_m1**	Gusb	ThermoFisher Scientific
**Mm00496696_g1**	S100A8	ThermoFisher Scientific
**Mm01324470_m1**	LCN2	ThermoFisher Scientific
