# Supplementary material for: A Computational Model of Bacterial Population Dynamics in Gastrointestinal Yersinia enterocolitica Infections in Mice
Source: Biology (Basel). 2022 Feb 12;11(2):297. doi: 10.3390/biology11020297 (PMC8869254; doi:10.3390/biology11020297)
Supplement: Supplementary file 1 [file biology-11-00297-s001.zip › Table S4 qRTPCR raw data.pdf]

Table S4. qRTPCR raw data.

Reg3γ

Mouse	Treat-ment	CP1 Gusb	CP2 Gusb	Mean CP Gusb	Eff. Gusb	Eff.$_{\bullet Gusb}^{Cp(contr.)-Cp(inf.)}$	CP1 Reg3γ	CP2 Reg3γ	Mean CP Reg3γ	Eff. Reg3γ	Eff.$_{\bullet Reg3\gamma}^{Cp(contr.)-Cp(inf.)}$	Fold change
**SPF M1**	control	24,55	25,28	24,92	1,907	0,914	14,37	14,77	14,57	1,884	0,964	1,054
**SPF M2**	control	25,32	25,26	25,29	1,907	0,718	15,87	15,87	15,87	1,884	0,423	0,590
**SPF M3**	control	25,18	25,21	25,20	1,907	0,763	15,21	15,26	15,24	1,884	0,633	0,829
**SPF M4**	control	24,08	24,23	24,16	1,907	1,493	12,76	12,75	12,76	1,884	3,043	2,038
**SPF M5**	control	24,29	24,36	24,33	1,907	1,338	14,12	14,14	14,13	1,884	1,274	0,952
**SPF M6**	infected	23,99	23,67	23,83	1,907	1,842	13,82	13,79	13,81	1,884	1,565	0,850
**SPF M7**	infected	25,20	25,34	25,27	1,907	0,727	14,40	14,51	14,46	1,884	1,037	1,426
**SPF M8**	infected	26,29	24,09	25,19	1,907	0,765	13,05	13,09	13,07	1,884	2,493	3,256
**SPF M9**	infected	24,13	24,16	24,15	1,907	1,503	13,29	13,25	13,27	1,884	2,196	1,461
**SPF M10**	infected	23,54	24,20	23,87	1,907	1,795	12,25	12,28	12,27	1,884	4,151	2,313
**GF M1**	control	27,74	26,43	27,09	1,907	0,225	21,86	21,84	21,85	1,884	0,010	0,043
**GF M2**	control	29,17	29,24	29,21	1,907	0,057	25,09	25,16	25,13	1,884	0,001	0,021
**GF M3**	control	26,99	27,10	27,05	1,907	0,231	20,99	20,98	20,99	1,884	0,017	0,072
**GF M4**	control	25,91	26,15	26,03	1,907	0,445	21,59	21,62	21,61	1,884	0,011	0,025
**GF M5**	control	26,96	26,96	26,96	1,907	0,244	22,48	22,52	22,50	1,884	0,006	0,026
**GF M6**	infected	26,99	26,98	26,99	1,907	0,240	20,48	20,51	20,50	1,884	0,023	0,094
**GF M7**	infected	28,01	28,46	28,24	1,907	0,107	21,98	21,96	21,97	1,884	0,009	0,083
**GF M8**	infected	26,23	25,98	26,11	1,907	0,424	18,98	18,90	18,94	1,884	0,061	0,143
**GF M9**	infected	26,58	26,57	26,58	1,907	0,313	20,68	20,73	20,71	1,884	0,020	0,063
**GF M10**	infected	26,91	27,50	27,21	1,907	0,208	21,18	21,21	21,20	1,884	0,015	0,070
**Myd88$^{-/-}$ M1**	control	25,33	25,80	25,57	1,907	0,601	17,87	17,88	17,88	1,884	0,119	0,198
**Myd88$^{-/-}$ M2**	control	24,92	24,91	24,92	1,907	0,914	16,73	16,70	16,72	1,884	0,248	0,271
**Myd88$^{-/-}$ M3**	control	24,90	24,98	24,94	1,907	0,900	17,79	17,69	17,74	1,884	0,129	0,144
**Myd88$^{-/-}$ M4**	control	25,82	25,82	25,82	1,907	0,510	18,81	18,83	18,82	1,884	0,065	0,128
**Myd88$^{-/-}$ M5**	control	24,68	24,73	24,71	1,907	1,047	17,13	17,39	17,26	1,884	0,175	0,168
**Myd88$^{-/-}$ M6**	infected	27,58	n.d.	27,58	1,907	0,164	18,71	18,74	18,73	1,884	0,069	0,424
**Myd88$^{-/-}$ M7**	infected	23,96	24,01	23,99	1,907	1,666	17,69	17,72	17,71	1,884	0,132	0,079
**Myd88$^{-/-}$ M8**	infected	24,31	24,34	24,33	1,907	1,338	16,54	16,56	16,55	1,884	0,275	0,206
**Myd88$^{-/-}$ M9**	infected	24,15	24,20	24,18	1,907	1,474	16,39	16,38	16,39	1,884	0,305	0,207
**Myd88$^{-/-}$ M10**	infected	24,10	24,05	24,08	1,907	1,572	16,32	16,46	16,39	1,884	0,304	0,194

LCN-2

Mouse	Treat-ment	CP1 Gusb	CP2 Gusb	Mean CP Gusb	Eff. Gusb	$Eff._{Gusb}^{Cp(contr.)-Cp(inf.)}$	CP1 LCN-2	CP2 LCN-2	Mean CP LCN-2	Eff. LCN-2	$Eff._{LCN-2}^{Cp(contr.)-Cp(inf.)}$	Fold change
**SPF M1**	control	24,37	24,44	24,41	1,820	1,095	30,58	30,67	30,63	1,591	0,870	0,795
**SPF M2**	control	24,86	24,84	24,85	1,820	0,839	31,90	32,00	31,95	1,591	0,470	0,561
**SPF M3**	control	25,21	25,16	25,19	1,820	0,686	30,60	30,60	30,60	1,591	0,880	1,283
**SPF M4**	control	24,07	24,12	24,10	1,820	1,318	28,70	28,71	28,71	1,591	2,122	1,610
**SPF M5**	control	24,25	24,24	24,25	1,820	1,205	29,68	29,81	29,75	1,591	1,309	1,087
**SPF M6**	infected	23,82	23,85	23,84	1,820	1,540	28,82	28,75	28,79	1,591	2,044	1,328
**SPF M7**	infected	25,08	25,14	25,11	1,820	0,718	30,32	30,48	30,40	1,591	0,966	1,346
**SPF M8**	infected	24,08	24,07	24,08	1,820	1,334	27,37	27,39	27,38	1,591	3,926	2,943
**SPF M9**	infected	24,06	24,12	24,09	1,820	1,322	28,22	28,24	28,23	1,591	2,645	2,001
**SPF M10**	infected	23,52	23,53	23,53	1,820	1,854	26,59	26,63	26,61	1,591	5,613	3,027
**GF M1**	control	26,3	26,46	26,38	1,820	0,335	35,32	35,08	35,20	1,591	0,104	0,310
**GF M2**	control	28,97	28,91	28,94	1,820	0,072	38,13	38,97	38,55	1,591	0,022	0,303
**GF M3**	control	26,90	27,01	26,96	1,820	0,238	37,59	37,61	37,60	1,591	0,034	0,143
**GF M4**	control	26,10	26,05	26,08	1,820	0,403	34,55	34,35	34,45	1,591	0,147	0,366
**GF M5**	control	26,89	26,84	26,87	1,820	0,251	35,63	35,88	35,76	1,591	0,080	0,320
**GF M6**	infected	26,81	26,84	26,83	1,820	0,257	35,73	35,83	35,78	1,591	0,079	0,309
**GF M7**	infected	27,78	27,79	27,79	1,820	0,145	35,75	35,48	35,62	1,591	0,086	0,593
**GF M8**	infected	25,99	25,95	25,97	1,820	0,429	33,13	33,06	33,10	1,591	0,276	0,644
**GF M9**	infected	26,46	26,35	26,41	1,820	0,330	33,62	33,89	33,76	1,591	0,203	0,615
**GF M10**	infected	26,90	26,97	26,94	1,820	0,241	35,49	35,58	35,54	1,591	0,089	0,370
**Myd88$^{-/-}$ M1**	control	24,64	24,63	24,64	1,820	0,954	32,01	32,85	32,43	1,591	0,376	0,394
**Myd88$^{-/-}$ M2**	control	24,36	24,35	24,36	1,820	1,128	31,63	31,60	31,62	1,591	0,549	0,487
**Myd88$^{-/-}$ M3**	control	24,79	24,77	24,78	1,820	0,874	32,49	32,52	32,51	1,591	0,363	0,416
**Myd88$^{-/-}$ M4**	control	25,92	25,95	25,94	1,820	0,438	33,71	33,57	33,64	1,591	0,215	0,490
**Myd88$^{-/-}$ M5**	control	23,91	23,89	23,90	1,820	1,481	30,67	30,79	30,73	1,591	0,829	0,559
**Myd88$^{-/-}$ M6**	infected	25,17	25,19	25,18	1,820	0,688	32,59	32,72	32,66	1,591	0,339	0,492
**Myd88$^{-/-}$ M7**	infected	23,87	23,97	23,92	1,820	1,464	30,58	30,63	30,61	1,591	0,878	0,600
**Myd88$^{-/-}$ M8**	infected	24,08	24,15	24,12	1,820	1,302	30,48	30,38	30,43	1,591	0,952	0,731
**Myd88$^{-/-}$ M9**	infected	24,30	24,48	24,39	1,820	1,105	30,33	30,58	30,46	1,591	0,941	0,852
**Myd88$^{-/-}$ M10**	infected	24,03	24,04	24,04	1,820	1,366	30,73	30,73	30,73	1,591	0,829	0,606

S100A8

Mouse	Treat-ment	CP1 Gusb	CP2 Gusb	Mean CP Gusb	Eff. Gusb	$Eff._{Gusb}^{Cp(contr.)-Cp(inf.)}$	CP1 S100A8	CP2 S100A8	Mean CP S100A8	Eff. S100A8	$Eff._{S100A8}^{Cp(contr.)-Cp(inf.)}$	Fold change
**SPF M1**	control	24,44	24,46	24,45	1,886	1,059	33,80	33,53	33,67	1,678	1,050	0,991
**SPF M2**	control	24,83	24,9	24,87	1,886	0,814	33,63	33,37	33,50	1,678	1,143	1,404
**SPF M3**	control	25,15	25,11	25,13	1,886	0,688	35,10	35,53	35,32	1,678	0,447	0,649
**SPF M4**	control	24,03	24,1	24,07	1,886	1,353	33,28	33,33	33,31	1,678	1,265	0,935
**SPF M5**	control	24,23	24,16	24,20	1,886	1,245	32,73	33,29	33,01	1,678	1,474	1,183
**SPF M6**	infected	23,80	23,77	23,79	1,886	1,616	30,62	30,47	30,55	1,678	5,278	3,267
**SPF M7**	infected	25,29	25,15	25,22	1,886	0,650	33,48	33,47	33,48	1,678	1,158	1,782
**SPF M8**	infected	24,24	24,24	24,24	1,886	1,210	25,07	25,16	25,12	1,678	87,721	72,471
**SPF M9**	infected	24,15	24,15	24,15	1,886	1,282	30,49	30,48	30,49	1,678	5,445	4,248
**SPF M10**	infected	23,72	23,5	23,61	1,886	1,805	28,66	28,70	28,68	1,678	13,859	7,677
**GF M1**	control	26,46	26,34	26,40	1,886	0,307	36,74	36,04	36,39	1,678	0,256	0,833
**GF M2**	control	29,29	28,98	29,14	1,886	0,054	39,85	39,09	39,47	1,678	0,052	0,960
**GF M3**	control	26,85	26,85	26,85	1,886	0,231	39,10	38,97	39,04	1,678	0,065	0,282
**GF M4**	control	26,14	26,08	26,11	1,886	0,370	38,36	38,52	38,44	1,678	0,089	0,240
**GF M5**	control	26,90	26,92	26,91	1,886	0,222	37,14	37,75	37,45	1,678	0,148	0,667
**GF M6**	infected	27,81	28,07	27,94	1,886	0,116	34,30	34,14	34,22	1,678	0,788	6,807
**GF M7**	infected	27,46	26,97	27,22	1,886	0,183	33,89	34,15	34,02	1,678	0,874	4,766
**GF M8**	infected	25,97	26,03	26,00	1,886	0,396	32,65	32,64	32,65	1,678	1,780	4,492
**GF M9**	infected	26,44	26,64	26,54	1,886	0,281	33,90	33,86	33,88	1,678	0,939	3,339
**GF M10**	infected	27,10	27,17	27,14	1,886	0,193	38,92	39,44	39,18	1,678	0,060	0,313
**$Myd88^{-/-}$ M1**	control	24,51	24,56	24,54	1,886	1,004	35,84	35,38	35,61	1,678	0,384	0,382
**$Myd88^{-/-}$ M2**	control	24,53	24,44	24,49	1,886	1,036	33,54	33,86	33,70	1,678	1,031	0,995
**$Myd88^{-/-}$ M3**	control	24,7	24,82	24,76	1,886	0,870	35,16	35,14	35,15	1,678	0,487	0,559
**$Myd88^{-/-}$ M4**	control	26,00	26,18	26,09	1,886	0,374	36,62	35,91	36,27	1,678	0,273	0,730
**$Myd88^{-/-}$ M5**	control	23,84	23,90	23,87	1,886	1,531	34,77	35,55	35,16	1,678	0,484	0,316
**$Myd88^{-/-}$ M6**	infected	25,15	25,17	25,16	1,886	0,675	35,86	35,14	35,50	1,678	0,406	0,601
**$Myd88^{-/-}$ M7**	infected	24,06	24,03	24,05	1,886	1,370	34,73	35,32	35,03	1,678	0,519	0,379
**$Myd88^{-/-}$ M8**	infected	24,01	24,03	24,02	1,886	1,392	32,78	32,68	32,73	1,678	1,703	1,224
**$Myd88^{-/-}$ M9**	infected	24,55	24,17	24,36	1,886	1,122	34,81	34,99	34,90	1,678	0,554	0,494
**$Myd88^{-/-}$ M10**	infected	24,44	24,34	24,39	1,886	1,101	34,77	34,96	34,87	1,678	0,564	0,513
