# Supplementary material for: A Computational Model of Bacterial Population Dynamics in Gastrointestinal Yersinia enterocolitica Infections in Mice
Source: Biology (Basel). 2022 Feb 12;11(2):297. doi: 10.3390/biology11020297 (PMC8869254; doi:10.3390/biology11020297)
Supplement: Supplementary file 1 [file biology-11-00297-s001.zip › Table S5 Data set used for calibration of model.pdf]

Table S5. Data set used to calibrate the model.

SPF wildtype/A0 coinfection

Time (hours post infection)	log10 CFU/g feces of Ye wildtype in lumen	log10 CFU/g feces of Ye YadA0 in lumen
24	3,08	3,22
24	3,39	2,41
24	2,05	2,16
24	2,05	2,11
24	4,18	2,37
24	3,56	2,44
24	4,21	2,49
24	3,59	2,73
24	3,72	3,22
24	2,05	2,60
24	3,28	2,86
24	4,16	3,44
24	3,89	2,97
24	5,47	3,85
24	4,02	3,35
24	4,65	3,88
24	5,44	3,16
48	6,21	5,17
48	6,76	5,79
48	5,73	2,05
48	5,84	2,05
48	7,04	4,78
48	6,61	4,58
48	5,85	5,48
48	6,64	3,86
48	4,53	2,05
48	5,90	4,15
48	3,50	2,52
48	6,72	5,64
48	6,06	2,07
48	7,10	5,02
48	6,85	4,05
48	7,54	2,79
48	6,19	2,05
72	6,44	5,13
72	7,16	6,10
72	6,37	3,53
72	6,77	3,19
72	7,50	3,55
72	6,93	2,61
72	7,23	5,58

72	7,12	3,09
72	5,80	3,79
72	7,01	3,59
72	5,43	2,05
72	7,13	2,05
72	5,72	2,05
72	7,84	5,02
72	7,60	6,66
72	6,35	2,05
72	5,91	2,05
120	7,18	3,07
120	6,99	3,00
120	6,76	3,03
120	6,74	3,29
120	6,11	2,66
120	6,34	2,87
120	7,65	2,85
120	6,56	3,34
120	5,81	3,02
120	7,35	3,29
120	4,98	3,28
120	6,77	3,48
120	6,85	3,13
120	7,51	3,01
120	7,38	2,79
120	6,12	3,19
120	5,62	2,96
168	5,56	2,27
168	6,22	2,98
168	6,46	2,36
168	6,32	2,96
168	7,24	2,45
168	6,96	2,87
168	6,32	2,62
168	7,00	2,39
168	5,07	3,09
168	7,17	3,05
168	4,44	2,86
168	6,29	2,68
168	6,60	3,17
168	7,26	2,93
168	7,24	2,54
168	4,84	2,98
240	5,60	2,05
240	6,33	2,05
240	6,62	2,05
240	5,47	2,05

240	6,55	2,05
240	5,91	2,05
240	6,51	2,05
240	6,30	2,05
240	6,94	2,05
240	5,12	2,05
240	5,53	2,05
240	6,77	2,05
240	6,11	2,05
240	6,85	2,05
240	4,84	2,05
336	6,42	2,05
336	6,65	2,05
336	7,16	2,05
336	5,88	2,05
336	6,01	2,05
336	6,31	2,05
336	7,04	2,05
336	6,64	2,05
336	7,68	2,05
336	5,01	2,05
336	6,43	2,05
336	6,07	2,05
336	6,39	2,05
336	6,16	2,05
336	6,75	2,05

SPF wildtype/A0 coinfection median

Time (hours post infection)	median of log10 CFU/g feces of Ye wildtype in lumen	median of log10 CFU/g feces of Ye YadA0 in lumen
24	3,72	2,86
48	6,21	4,05
72	6,93	3,53
120	6,76	3,03
168	6,39	2,87
240	6,30	2,05
336	6,42	2,05

SPF wildtype/T3S0 coinfection

Time (hours post infection)	log10 CFU/g feces of Ye wildtype in lumen	log10 CFU/g feces of Ye T3S0 in lumen
24	2,66	2,66
24	2,05	2,05
24	5,67	4,01
24	4,14	2,84
24	4,45	2,87
24	3,61	2,46
24	4,26	2,05
24	3,91	2,05
24	5,35	3,47
24	4,00	2,05
24	4,78	3,00
24	3,71	2,05
24	3,70	3,22
24	4,75	2,05
48	4,56	2,05
48	3,37	2,05
48	6,15	2,05
48	6,84	2,05
48	6,58	2,11
48	6,12	2,05
48	5,94	2,05
48	6,17	3,23
48	7,24	5,02
48	6,53	2,50
48	6,26	2,45
48	6,75	2,21
48	6,37	2,05
48	6,65	2,05
72	6,91	2,05
72	3,76	2,05
72	7,06	2,05
72	6,63	2,73
72	6,67	3,08
72	6,35	2,05
72	6,70	2,05
72	6,08	2,88
72	7,35	2,86
72	6,83	2,63

72	6,73	2,05
72	6,78	2,05
72	7,04	3,04
72	7,31	2,05
120	6,30	2,85
120	4,34	2,57
120	7,13	3,30
120	5,31	3,23
120	5,78	3,14
120	5,90	3,05
120	6,99	3,03
120	6,77	3,57
120	6,70	3,21
120	5,73	3,21
120	7,14	3,41
120	6,90	3,03
120	6,10	2,94
120	6,80	3,40
168	5,24	3,31
168	4,01	2,46
168	6,56	2,89
168	6,78	2,98
168	7,07	3,13
168	6,92	2,69
168	5,51	2,91
168	6,44	3,43
168	6,29	3,08
168	7,13	2,86
168	6,41	2,74
168	4,95	3,08
168	7,13	3,17
240	5,85	2,05
240	3,54	2,05
240	5,71	2,05
240	5,72	2,05
240	5,56	2,05
240	5,62	2,05
240	6,42	2,05
240	5,91	2,05
240	6,45	2,05
240	6,15	2,05
336	6,08	2,05
336	4,29	2,05

336	7,03	2,05
336	6,08	2,05
336	6,32	2,05
336	6,69	2,05
336	6,50	2,05
336	6,18	2,05
336	6,14	2,05
336	6,32	2,05

SPF wildtype/T3S0 coinfection median

Time (hours post infection)	median of log10 CFU/g feces of Ye wildtype in lumen	median of log10 CFU/g feces of Ye T3S0 in lumen
24	4,07	2,56
48	6,32	2,05
72	6,76	2,05
120	6,50	3,18
168	6,44	2,98
240	5,79	2,05
336	6,25	2,05

GF wildtype/A0 coinfection

Time (hours post infection)	log10 CFU/g feces of Ye wildtype in lumen	log10 CFU/g feces of Ye YadA0 in lumen
24	8,47	8,52
24	8,58	8,67
24	8,51	8,86
24	8,37	8,49
24	8,60	8,64
24	8,80	8,63
24	8,68	8,16
24	8,67	8,85
24	8,38	8,31
24	8,53	8,98
48	8,96	8,77
48	8,78	8,78
48	8,69	8,90
48	8,96	9,18
48	8,53	8,52
48	8,99	9,00
48	8,68	8,92
48	9,07	8,41
48	8,27	8,67
48	8,55	8,51

72	8,38	8,79
72	8,70	8,71
72	8,56	8,68
72	8,32	8,40
72	8,73	8,74
72	8,41	8,05
72	8,73	8,68
72	9,16	8,92
72	8,53	8,74
72	8,68	8,35
120	8,40	8,48
120	8,88	8,84
120	8,72	8,55
120	8,83	8,68
120	8,78	8,41
120	8,63	8,73
120	8,40	8,38
120	8,82	8,98
120	8,25	8,27
120	8,64	8,94
168	8,42	8,28
168	8,72	8,82
168	8,64	8,99
168	8,77	8,67
168	9,02	9,14
168	8,66	8,95
168	8,53	8,99
168	8,71	8,62
168	8,87	8,74
168	8,65	8,61
240	8,67	8,68
240	9,17	8,81
240	9,24	9,12
240	8,88	8,86
240	8,23	8,23
240	8,78	8,98
240	9,02	8,92
240	8,87	9,11
240	8,75	8,92
240	8,75	9,02
336	8,66	8,59
336	9,00	9,14
336	8,23	8,59
336	8,80	8,76
336	8,81	8,71
336	8,36	8,59
336	8,82	8,52

336	8,62	8,64
336	8,30	8,54
336	8,36	8,18

GF wildtype/A0 coinfection median

Time (hours post infection)	median of log10 CFU/g feces of Ye wildtype in lumen	median of log10 CFU/g feces of Ye YadA0 in lumen
24	8,56	8,64
48	8,74	8,78
72	8,62	8,70
120	8,68	8,62
168	8,69	8,78
240	8,83	8,92
336	8,64	8,59

GF wildtype/T3S0 coinfection

Time (hours post infection)	log10 CFU/g feces of Ye wildtype in lumen	log10 CFU/g feces of Ye T3S0 in lumen
24	8,91	8,39
24	8,35	8,45
24	8,81	8,71
24	8,99	8,66
24	8,98	8,77
24	9,28	9,05
24	9,12	8,92
24	8,53	8,23
24	8,37	8,21
24	8,96	8,66
48	9,46	8,51
48	8,89	8,82
48	9,27	9,02
48	9,10	8,88
48	9,38	8,87
48	9,13	8,39
48	8,85	8,40
48	9,26	9,02
48	9,19	8,55
48	9,19	9,01
72	8,09	7,70
72	8,83	8,33
72	8,87	8,59
72	9,25	8,81
72	8,89	8,52

72	8,81	8,55
72	9,12	8,70
72	8,86	8,78
72	8,63	8,58
72	9,09	8,81
120	8,83	8,39
120	9,01	8,75
120	8,94	8,59
120	8,79	8,48
120	8,96	8,70
120	9,04	8,97
120	9,20	8,83
120	9,44	9,14
120	8,94	8,57
120	9,11	8,65
168	9,06	8,69
168	9,11	8,77
168	8,95	8,42
168	9,01	8,71
168	9,02	8,53
168	8,83	8,10
168	8,99	8,59
168	8,89	8,46
168	8,90	8,49
240	9,23	8,53
240	8,90	8,51
240	8,25	8,04
240	8,88	8,65
240	8,18	7,95
240	8,19	7,72
240	8,32	8,02
240	8,37	8,09
240	8,23	7,88
336	9,38	8,76
336	8,62	8,49
336	8,20	7,79
336	8,88	8,38
336	7,73	7,21
336	8,34	8,11
336	7,95	7,83
336	8,31	7,83
336	8,06	7,48

GF wildtype/T3S0 coinfection median

Time (hours post infection)	median of log10 CFU/g feces of Ye wildtype in lumen	median of log10 CFU/g feces of Ye T3S0 in lumen
24	8,94	8,66
48	9,19	8,85
72	8,87	8,59
120	8,99	8,68
168	8,99	8,53
240	8,32	8,04
336	8,31	7,83

MyD88$^{-/-}$ wildtype/A0 coinfection

Time (hours post infection)	log10 CFU/g feces of Ye wildtype in lumen	log10 CFU/g feces of Ye YadA0 in lumen
16	4,90	4,18
16	5,26	4,06
16	4,76	3,65
16	4,50	4,29
16	4,71	4,38
16	1,79	2,07
16	4,14	3,90
16	3,88	2,76
16	5,03	4,63
16	3,45	2,81
24	2,64	2,34
24	3,17	3,18
24	2,74	1,74
24	3,62	3,13
24	3,76	2,05
24	3,77	2,24
24	4,14	2,27
24	3,06	2,46
24	5,23	3,23
24	3,97	3,27
40	3,55	4,17
40	5,25	5,73
40	3,18	1,79
40	5,63	4,25
40	4,62	1,63
40	5,63	1,79
40	4,32	2,28
40	3,90	3,14

40	5,54	5,35
40	3,27	2,42
48	3,74	4,84
48	5,27	5,68
48	5,67	1,79
48	6,33	3,62
48	6,01	1,79
48	6,27	1,53
48	4,51	1,83
48	3,34	2,63
48	4,73	6,55
48	3,10	2,12

MyD88$^{-/-}$ wildtype/A0 coinfection median

Time (hours post infection)	median of log10 CFU/g feces of Ye wildtype in lumen	median of log10 CFU/g feces of Ye YadA0 in lumen
16	4,61	3,98
24	3,69	2,40
40	4,47	2,78
48	5,00	2,38

MyD88$^{-/-}$ wildtype/T3S0 coinfection

Time (hours post infection)	log10 CFU/g feces of Ye wildtype in lumen	log10 CFU/g feces of Ye T3S0 in lumen
16	3,45	3,20
16	3,46	3,39
16	3,16	4,03
16	4,52	3,81
16	2,65	2,65
16	3,23	3,58
16	3,61	3,17
16	3,99	3,42
16	3,17	3,58
16	3,42	3,79
24	2,24	2,24
24	2,80	4,48
24	5,98	4,38
24	2,24	2,24
24	1,94	1,94
24	3,42	2,24
24	5,11	3,04

24	3,07	2,04
24	3,65	2,51
24	2,24	2,24
40	2,71	3,19
40	4,59	5,52
40	6,52	5,66
40	4,57	3,57
40	5,32	3,20
40	4,63	3,58
40	5,94	4,59
40	3,76	3,31
40	3,86	3,00
40	4,25	3,56
48	2,96	3,36
48	3,88	3,65
48	6,61	5,20
48	5,34	2,24
48	5,21	3,20
48	4,25	4,03
48	4,72	4,34
48	3,24	3,16
48	3,55	1,95
48	3,64	3,19

MyD88$^{-/-}$ wildtype/T3S0 coinfection median

Time (hours post infection)	median of log10 CFU/g feces of Ye wildtype in lumen	median of log10 CFU/g feces of Ye T3S0 in lumen
16	3,44	3,50
24	2,94	2,24
40	4,58	3,57
48	4,07	3,28

CFU values at detection limit are labeled.
