# Supplementary figures and images for: A Computational Model of Bacterial Population Dynamics in Gastrointestinal Yersinia enterocolitica Infections in Mice
Source: Biology (Basel). 2022 Feb 12;11(2):297. doi: 10.3390/biology11020297 (PMC8869254; doi:10.3390/biology11020297)

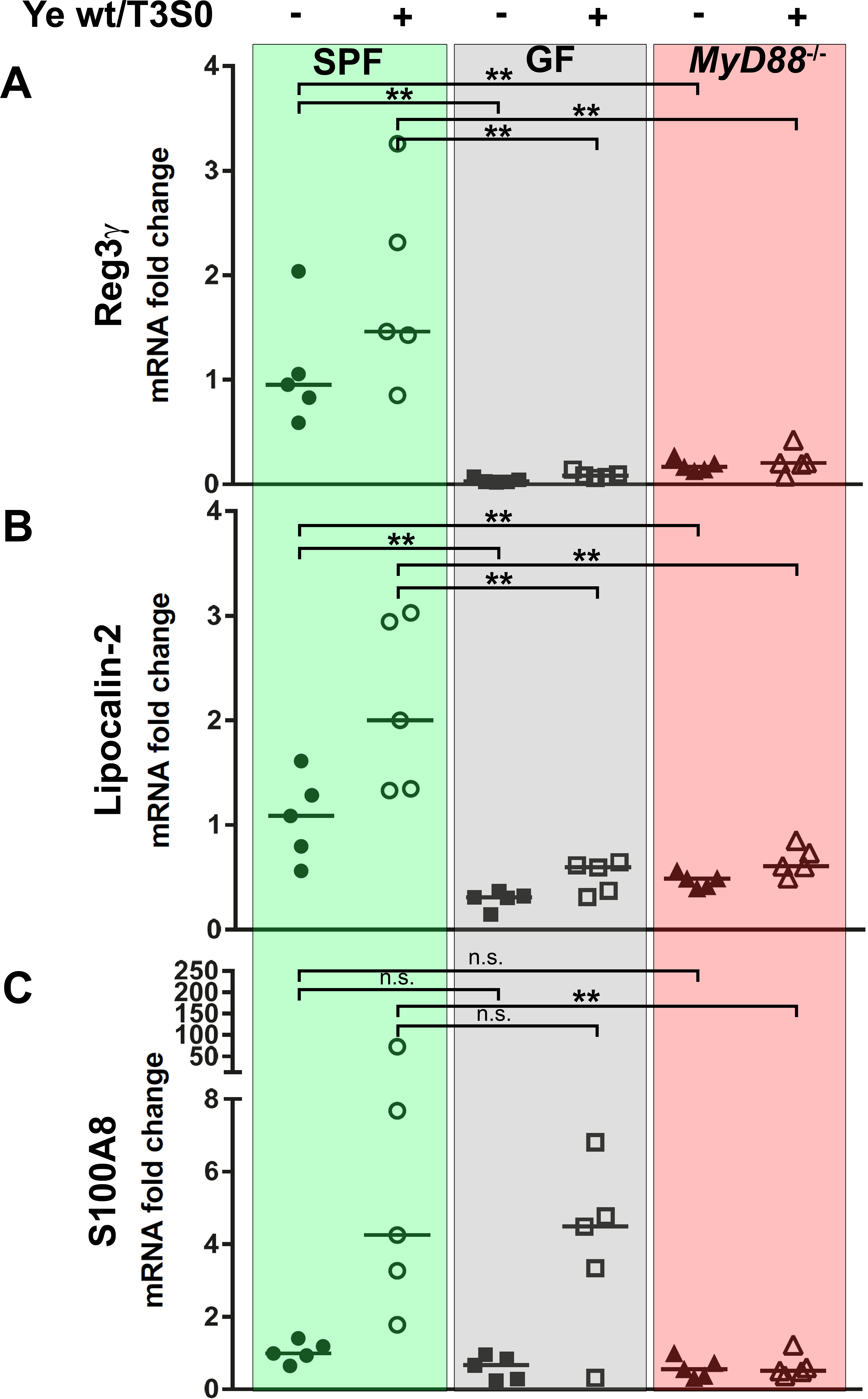

Supplement: Supplementary file 1 [file biology-11-00297-s001.zip › Figure S2 Geißert et al Immunparameter.png]

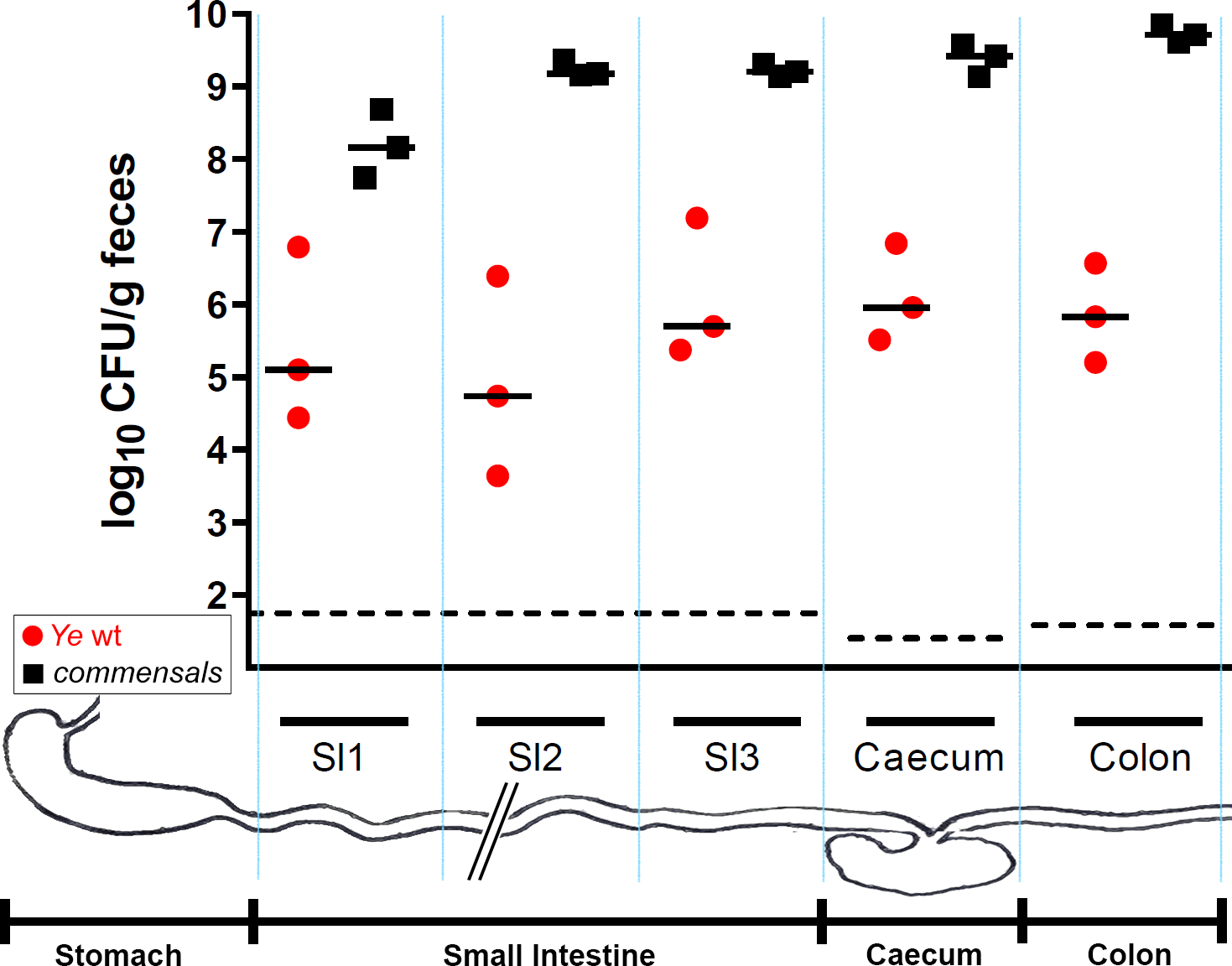

Supplement: Supplementary file 1 [file biology-11-00297-s001.zip › Figure S3 Geißert et al Localisation of Ye in the GIT.png]

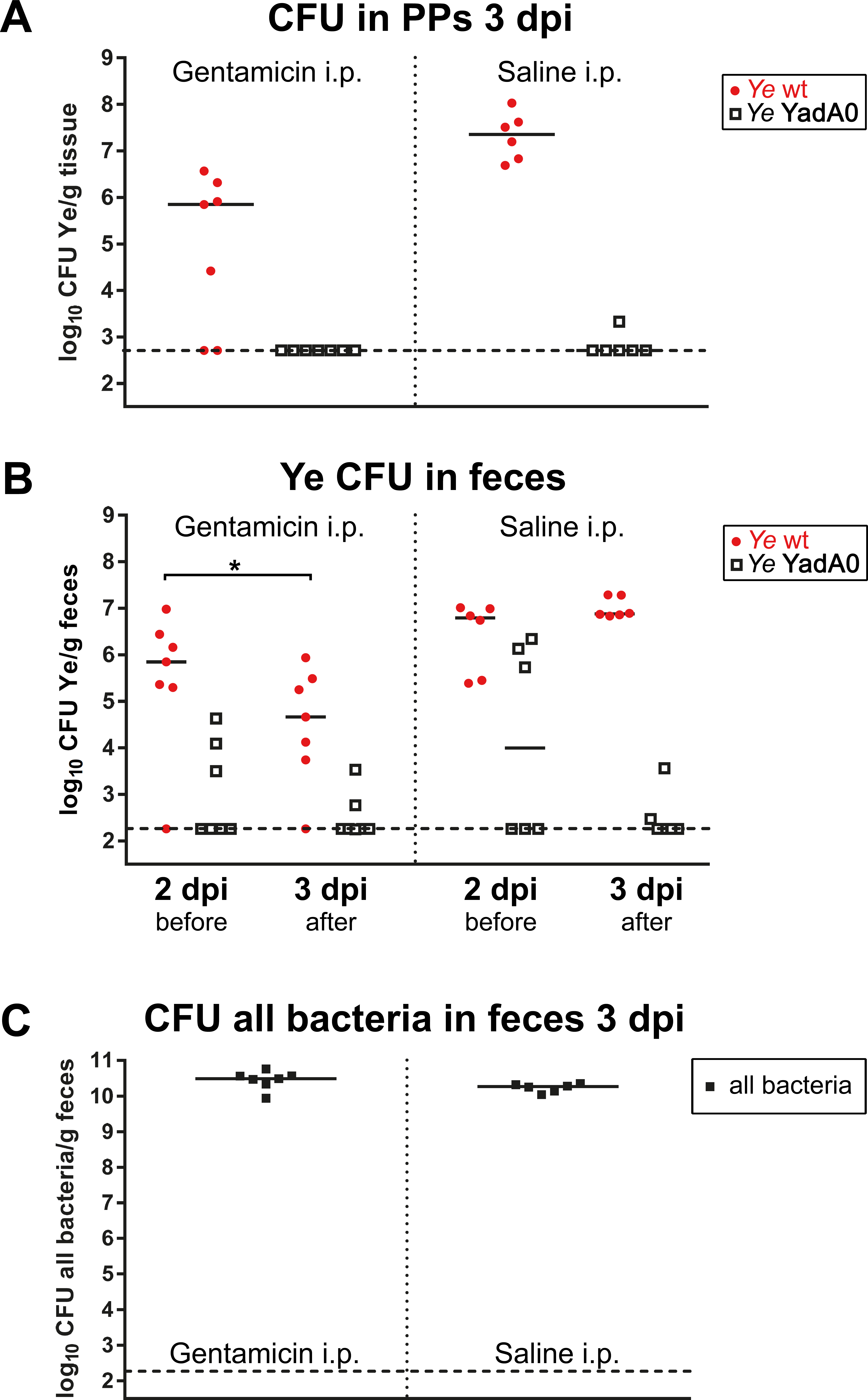

Supplement: Supplementary file 1 [file biology-11-00297-s001.zip › Figure S4 Geißert et al Genta Clean.png]

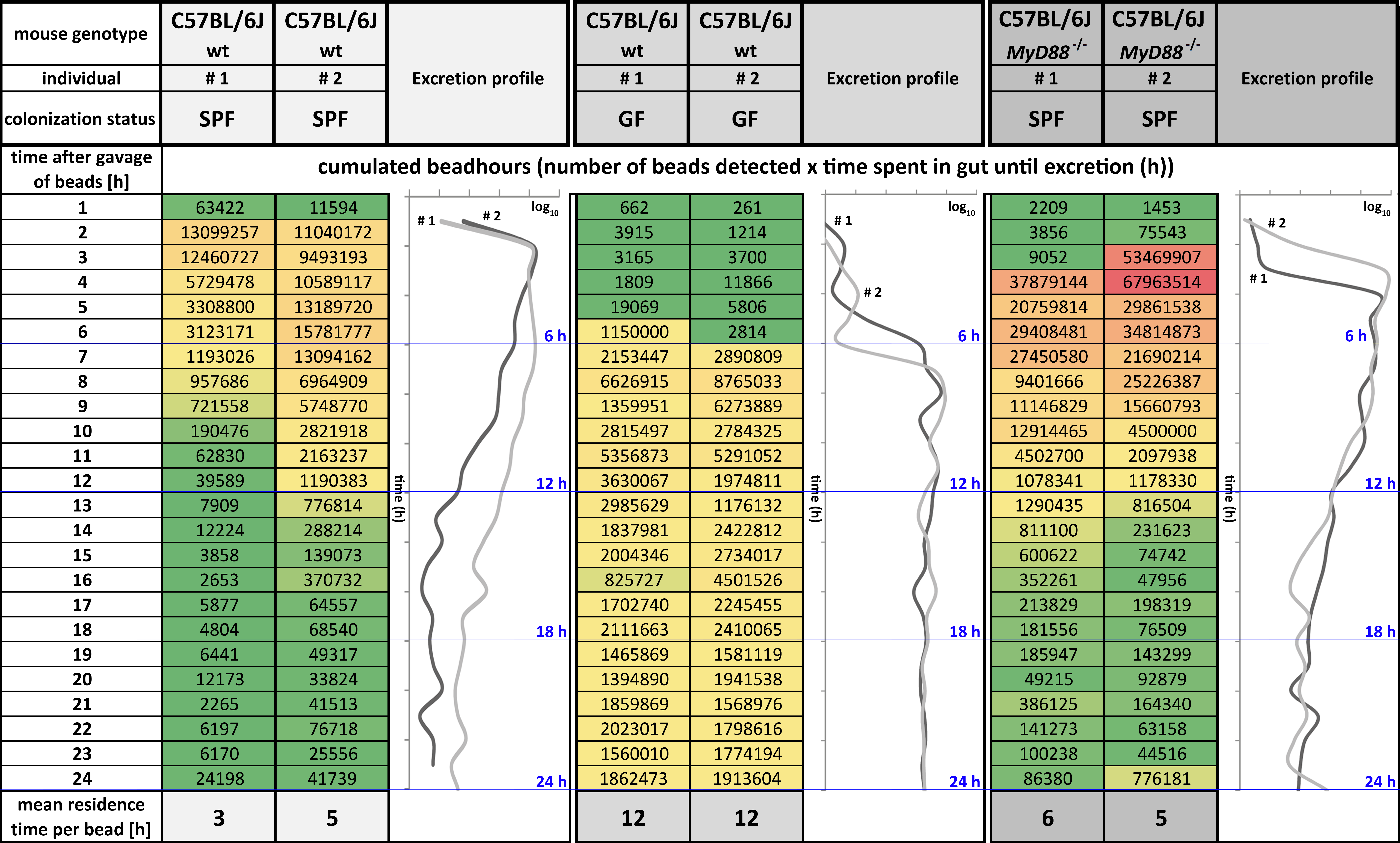

Supplement: Supplementary file 1 [file biology-11-00297-s001.zip › Figure S5 Geißert et al GIT passage.png]
